# Estimating the Health Effects of Adding Bicycle and Pedestrian Paths at the Census Tract Level: Multiple Model Comparison

**DOI:** 10.2196/37379

**Published:** 2022-08-24

**Authors:** Ross Gore, Christopher J Lynch, Craig A Jordan, Andrew Collins, R Michael Robinson, Gabrielle Fuller, Pearson Ames, Prateek Keerthi, Yash Kandukuri

**Affiliations:** 1 Virginia Modeling Analysis and Simulation Center Old Dominion University Suffolk, VA United States; 2 Engineering Management & Systems Engineering Old Dominion University Norfolk, VA United States; 3 Department of Biomedical Engineering University of Virginia Charlottesville, VA United States; 4 Department of Economics Chapman University Orange, CA United States; 5 Hampton Roads Biomedical Research Consortium Norfolk, VA United States

**Keywords:** bicycle paths, pedestrian paths, bicycling, walking, diabetes, high blood pressure, physical health, factor analysis, digital neighborhoods, data analysis

## Abstract

**Background:**

Adding additional bicycle and pedestrian paths to an area can lead to improved health outcomes for residents over time. However, quantitatively determining which areas benefit more from bicycle and pedestrian paths, how many miles of bicycle and pedestrian paths are needed, and the health outcomes that may be most improved remain open questions.

**Objective:**

Our work provides and evaluates a methodology that offers actionable insight for city-level planners, public health officials, and decision makers tasked with the question “To what extent will adding specified bicycle and pedestrian path mileage to a census tract improve residents’ health outcomes over time?”

**Methods:**

We conducted a factor analysis of data from the American Community Survey, Center for Disease Control 500 Cities project, Strava, and bicycle and pedestrian path location and use data from two different cities (Norfolk, Virginia, and San Francisco, California). We constructed 2 city-specific factor models and used an algorithm to predict the expected mean improvement that a specified number of bicycle and pedestrian path miles contributes to the identified health outcomes.

**Results:**

We show that given a factor model constructed from data from 2011 to 2015, the number of additional bicycle and pedestrian path miles in 2016, and a specific census tract, our models forecast health outcome improvements in 2020 more accurately than 2 alternative approaches for both Norfolk, Virginia, and San Francisco, California. Furthermore, for each city, we show that the additional accuracy is a statistically significant improvement (*P*<.001 in every case) when compared with the alternate approaches. For Norfolk, Virginia (n=31 census tracts), our approach estimated, on average, the percentage of individuals with high blood pressure in the census tract within 1.49% (SD 0.85%), the percentage of individuals with diabetes in the census tract within 1.63% (SD 0.59%), and the percentage of individuals who had >2 weeks of poor physical health days in the census tract within 1.83% (SD 0.57%). For San Francisco (n=49 census tracts), our approach estimates, on average, that the percentage of individuals who had a stroke in the census tract is within 1.81% (SD 0.52%), and the percentage of individuals with diabetes in the census tract is within 1.26% (SD 0.91%).

**Conclusions:**

We propose and evaluate a methodology to enable decision makers to weigh the extent to which 2 bicycle and pedestrian paths of equal cost, which were proposed in different census tracts, improve residents’ health outcomes; identify areas where bicycle and pedestrian paths are unlikely to be effective interventions and other strategies should be used; and quantify the minimum amount of additional bicycle path miles needed to maximize health outcome improvements. Our methodology shows statistically significant improvements, compared with alternative approaches, in historical accuracy for 2 large cities (for 2016) within different geographic areas and with different demographics.

## Introduction

The addition of bicycle and pedestrian paths to an area is a theoretically valuable resource for city-level planners, public health officials, and decision makers to increase physical activity and improve health outcomes. Most existing research has found a negative association between the prevalence of bicycle and pedestrian paths and poor health outcomes (ie, diabetes, stroke, obesity, heart disease, high blood pressure, and ailments to physical and mental health) [1-10].

### Objectives

Our objective is to provide and evaluate a methodology for officials addressing the question “To what extent will adding specified bicycle and pedestrian path mileage to a census tract improve residents’ health outcomes over time?” The methodology we propose uses factor analysis to filter and organize variables from publicly available data sets at the census tract level within a given city. The data sets included (1) the US Census [11], (2) the American Communities Survey (ACS) [12], (3) Centers for Disease Control and Prevention (CDC) 500 Cities project data [13], (4) municipality data [14,15], and (5) the GPS walking, running, and cycling tracking social network app, Strava [16,17].

The result of this analysis is a city-specific factor model describing the relationship among variables related to individuals, bicycling and walking behaviors, and health outcomes. Then, the factor model, built using past data, is used in an algorithm to predict the extent to which adding a future specified number of bicycle and pedestrian path miles to a certain location in the city quantitatively impacts certain health outcomes.

### Background

We are not aware of any other applications of factor analysis to develop predictive algorithms related to the placement and efficacy of bicycle and pedestrian paths with respect to health outcomes. However, there are researchers who approach bicycle and pedestrian path planning from a similar perspective. Smith and Haghani [18] proposed an approach that adds bicycle and pedestrian paths within a city such that the length of the average trip within the bicycle and pedestrian path network is minimized, and the level of service of the bicycle and pedestrian paths is maximized. Mesbah et al [19] explored the addition of bicycle and pedestrian paths within a city by identifying locations that minimized the total travel time of automobiles within the city. Researchers assume that bicycle and pedestrian paths take road space from cars. Although this assumption may occasionally be true, in most instances, bicycle and pedestrian paths narrow car lanes but do not reduce the total number available. Duthie and Unnikrishnan [20] identified instances within a city where the addition of bicycle and pedestrian paths maximized the connectivity of the existing bicycle and pedestrian path network. This approach ignores the use of the current bicycle and pedestrian path network and aims to “open up” as many new routes as possible regardless of current demand [21].

Although they are not prevalent in identifying bicycle and pedestrian path placement, optimization techniques have also been explored for choosing existing routes rather than developing new ones. Allen-Munley et al [22] developed a model that rates bicycle routes based on predictions of injury severity [18]. Other researchers have proposed allowing users to select multiple criteria and then eliminate certain routes (ie, steep slopes and heavy traffic) before providing a set of suggestions [23,24]. More recently, researchers have explored the use of multiobjective optimization as a means of retrofitting the existing cycling infrastructure for commuter cyclists. The objective of the formulation is to maximize the network for a number of different criteria, including accessibility, minimization of the number of intersections, maximization of bicycle level of service, and minimization of total construction cost subject to space-time constraints and monetary budget [25-27].

Ospina et al [28] addressed a similar problem but framed it as a maximal covering bicycle network design problem. The maximal covering bicycle network design problem involves making investment decisions to build a cycling network aimed at maximizing the coverage of cyclists while maintaining a minimum total network cost. The derived network is subject to budget and accounts for the entire connectivity and directness as fundamental bicycle network design criteria. This approach focuses only on the network and not on the health outcomes. There is no consideration of the extent to which each path in the network improves any health outcome within an area.

It is important to note that there are arguments against defining the placement of bicycle and pedestrian paths as a systems engineering problem. Szimba and Rothengatter [29] demonstrated that interdependencies between infrastructure projects can create cost incentives to place bicycle and pedestrian paths in certain areas, even if the payoff of the addition is not optimal with respect to the use, connectivity, or health benefits of the bicycle and pedestrian path. In addition, in areas where congestion and the propagation of congestion along bicycle and pedestrian paths occur, researchers have demonstrated that optimizing the use and distance of bicycle and pedestrian paths would only exacerbate traffic within the network and not produce effective results [30-32].

Furthermore, significant work has been conducted to estimate demand [33,34] and understand why people choose to use bicycle and pedestrian paths [35-40]. Our work also considers motivation related to bicycle and pedestrian path use but does not directly attempt to optimize bicycle and pedestrian path use. We made this design choice because adding bicycle and pedestrian paths based only on the existing demand can lead to a chicken-and-egg problem. Here, areas with advanced bicycle and pedestrian path infrastructure improve, and areas without bicycle and pedestrian path infrastructure are neglected. These dynamics can create inequitable living conditions and produce enormous health and environmental disparities within a city [41].

In summary, the algorithm used in this study is unique from previous approaches used for estimating demand, evaluating network efficacy, and optimizing the placement of bicycle and pedestrian paths. The problem examined here focuses on understanding what health outcomes can be improved by adding bicycle and pedestrian paths, in which census tracts will adding bicycle and pedestrian paths improve health outcomes the most, and finally, how many miles of bicycle and pedestrian paths within a given census tract need to be added to have an impact on the residents’ health outcomes.

The remainder of this paper is organized as follows. First, we review the data and methods used in our approach to construct city-specific models. Next, we apply the approach to two different cities: Norfolk, Virginia, and San Francisco, California. We then evaluate our approach for the 2 different cities. In the evaluation, our approach was tested against 2 alternate approaches for predicting improvements in health outcomes by adding bicycle and pedestrian paths. The evaluation shows that our approach offers more accurate predictions than both alternatives and that the superior difference in accuracy is statistically significant (*P*<.001 in all cases). Finally, we identify several limitations to our work and threats to its validity and review other avenues of related research.

## Methods

### Ethical Considerations

Our work uses publicly-available data related to urban infrastructure and resident demographics and health outcomes. The data sets reflect aggregate variables measured at the census tract level of a city and do not contain any personally identifiable information. Therefore, they do not involve human subjects as defined by federal regulations and their use does not require ethics board review or approval [42].

### Data Sets

#### Overview

**Figure 1:**
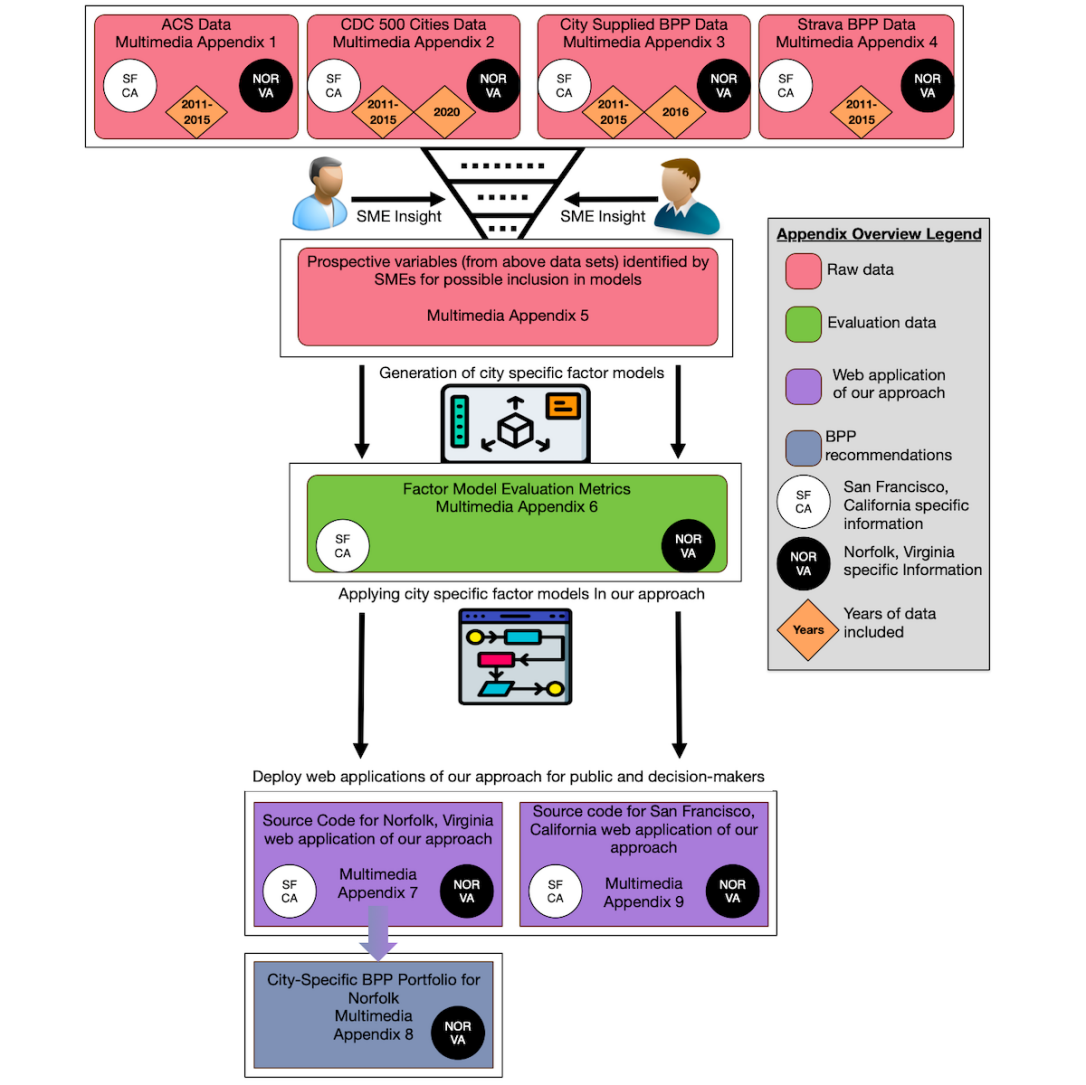
An overview of the data sets and other supplementary materials supplied in the multimedia appendices. ACS: American Communities Survey; BPP: bicycle and pedestrian path; CDC: Centers for Disease Control and Prevention; NOR: Norfolk; SF: San Francisco; SME: subject matter expert.

Our approach to modeling the health effects of adding bicycle and pedestrian paths at the census tract level uses data from (1) census tract boundaries used in the US Census [11]; (2) demographic variables from the ACS [12]; (3) census tract–level estimates for health outcomes, health statuses, healthy behaviors, and disease prevention from the CDC [13]; (4) bicycle and pedestrian path location and use data from Norfolk, Virginia, and San Francisco, California [14,15]; and (5) bicycle and pedestrian path use data from the GPS walking, running, and cycling tracking social network app, Strava. Combining these data sets resulted in >400 variables for each census tract in Norfolk, Virginia, and San Francisco, California [16,17]. An overview of all the data sets and other supplementary materials supplied in the multimedia appendices of this paper is shown in Figure 1.

#### US Census and ACS

Census tracts are small, contiguous, and relatively permanent statistical subdivisions of a county or an equivalent entity. The populations in census tracts vary from 1200 to 8000. Census tracts provide a stable geographic unit for statistical analysis in the US Census and ACS [43].

The ACS is an ongoing national survey that samples a subset of individuals within the same geographic areas in the US Census. Using the same questions, data were collected each month throughout the year. In contrast, the US Census provides a more comprehensive sample of individuals in the United States, collecting data from more individuals during a particular period (March to August) but administered only once every 10 years. A metaphor helps elucidate the differences between the 2 surveys. The US Census serves as a high-resolution photograph of the US population once every 10 years, whereas the ACS serves as many low-resolution continually updated videos over the same period [43]. Multimedia Appendix 1 provides the data included in the ACS for this study.

#### CDC 500 Cities Project

The census tract–level estimates and methodology for estimating health outcomes, health statuses, healthy behaviors, and disease prevention are provided by the CDC 500 Cities project. The 500 Cities project is a collaboration between the CDC and the Robert Wood Johnson Foundation. The small area estimates provided by the project allow policymakers and local health departments to better understand the burden and geographic distribution of health-related variables in their jurisdictions and assist them in planning public health interventions [13]. The data included in the CDC 500 Cities project for this study are provided in Multimedia Appendix 2.

#### City-Supplied Bicycle and Pedestrian Path Data

The bicycle and pedestrian path data for Norfolk, Virginia, and San Francisco, California include the latitude and longitude location of bicycle lanes, routes, and paths built and maintained in each city. Bicycle use data were taken from bicycle counters used in each city [14,15]. The data included from Norfolk, Virginia, and San Francisco, California, for this study are provided in Multimedia Appendix 3.

#### Strava Data

We used the Strava Metro rollup data set for Norfolk, Virginia, and San Francisco, California. This data set contains walking, running, and bicycling activity counts per road segment for a given year. These counts can then be aggregated at the census tract level. The road count segment is referred to as edge within Strava. Each edge is associated with a latitude and longitude bounding box using the Strava application programming interface [16,17]. The Strava data for Norfolk, Virginia, and San Francisco, California for this study are provided in Multimedia Appendix 4. There are limitations to using the Strava data, which we describe in the *Discussion* section.

### Data Selection

**Figure 2:**
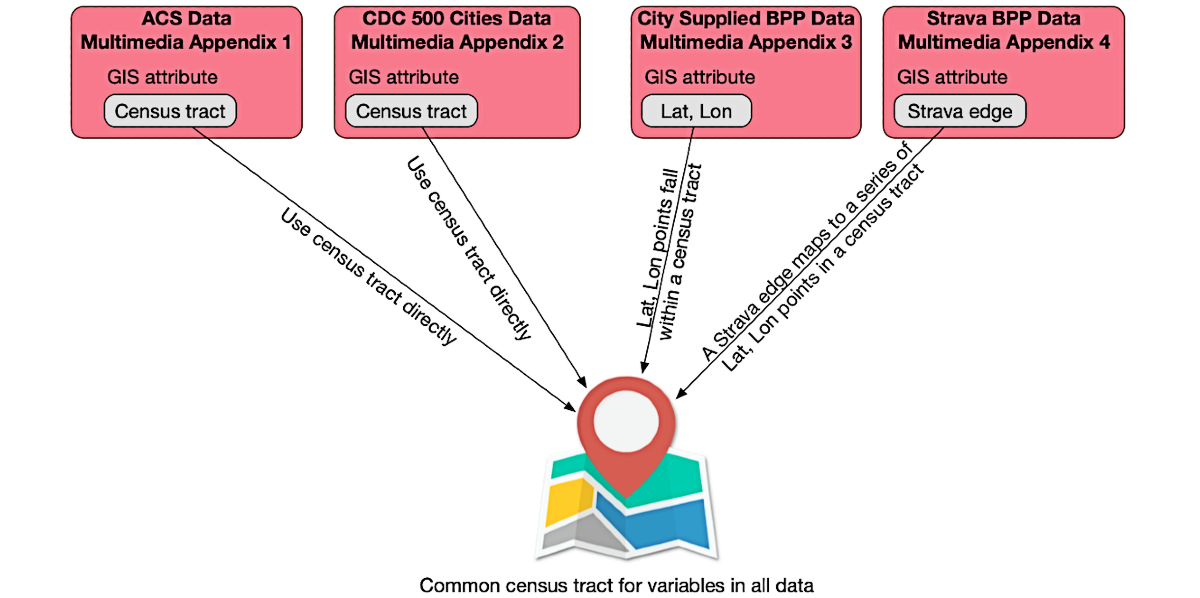
The approach to joining together the data sets at the census tract level. ACS: American Communities Survey; BPP: bicycle and pedestrian path; CDC: Centers for Disease Control and Prevention; GIS: Geographical Information System.

Our data set included a wide range of variables collected from multiple sources. From this data set, we selected a subset of the variables that individuals with domain expertise identified as possibly contributing to the use of bicycle and pedestrian paths and the impact of bicycle and pedestrian paths on health outcomes when additional mileage was added to a geographic area (ie, census tract). The expertise of these individuals spanned social work, health science and nutrition, community health, public health, and transportation. Textbox 1 shows the categories of variables identified by domain experts for each census tract in Norfolk, Virginia, and San Francisco, California. Multimedia Appendix 5 provides the list of observed variables for each category. These variables can be combined using common Geographical Information System attributes to align data at the census tract level. The approach for joining these data together at the census travel level is shown in Figure 2.

**The categories of variables from our data sets that are included in our factor analysis for Norfolk, Virginia, and San Francisco, California.**

**Data set and variable category**

- American Communities Survey
  Race
  Educational attainment
  Employment status
  Income and benefits
  Marital status
  Sex and age
  Commuting to work
  Citizenship
  Health insurance
  Occupation
  Household by type
  Relationship
- Centers for Disease Control and Prevention 500 Cities project
  Health outcomes
  Health risk behaviors
  Prevention
  Health status
- City Bicycle and Pedestrian Path data
  Bicycle and Pedestrian Path use data
  Bicycle and Pedestrian Path mileage data
- Strava Bicycle and Pedestrian Path data
  Bicycle and Pedestrian Path use data

### Factor Analysis

#### Overview

Next, we applied factor analysis to reduce these observed variables into latent variables (ie, factors). Factor analysis generates a model that measures how changes in one factor predict changes in another by reducing a large number of observed variables to a handful of comprehensible underlying factors. The result is an interpretable and actionable model of concepts that are otherwise difficult to measure [44].

The Honesty-Humility (H), Emotionality (E), Extraversion (X), Agreeableness (A), Conscientiousness (C), and Openness to Experience (O) 6D model of the human personality structure is a widely known result of the application of factor analysis. The ability of factor analysis to reduce the many observed variables related to personality into 6 distinct factors has pushed the state of the art in psychological research [45]. Our goal of applying factor analysis was similar.

We applied exploratory factor analysis (EFA) to filter the observed variables from the data described in Textbox 1 and reduced them into a model composed of factors that include residents’ (1) demographics and background characteristics (DBC), (2) health, and (3) bicycling and pedestrian habits (BPH). Using this model, we can understand how changes in one factor predict changes in others.

#### EFA Summary

In our approach, EFA was used to fit a factor model. Before the EFA began, data corresponding to half of a given city’s census tracts were selected at random. In the application of our approach, data from 2011 to 2015 were used. Then, using these data, an EFA model was fitted.

**Figure 3:**
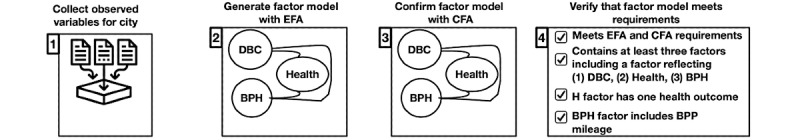
The process of generating a factor model for a city and verifying that it meets our defined restrictions. BPH: bicycling and pedestrian habits; BPP: bicycle and pedestrian path; CFA: confirmatory factor analysis; DBC: demographics and background characteristics; EFA: exploratory factor analysis.

Figure 3 shows the fitting of the model using EFA. The process is iterative, and each iteration comprises 3 stages. Figure 3A shows the observed variables that underwent analysis for a given iteration. These observed variables are organized into a number of factors that optimize the fit of the model in Figure 3B. The optimization constructs a model with the minimum number of factors such that the observed variables associated with each factor have maximum commonality with one another and minimal commonality with the observed variables in all other factors. Commonality reflects the amount of variance an observed variable shares with other variables in a factor [44,46].

Finally, the model was assessed. The assessment tests if all factors are composed of variables with high communality (>0.5) with respect to the factor they are associated with and low communality (<0.5) with all other factors. If this is true, the process terminates. Otherwise, variables that do not meet the communality requirement are discarded and the process is repeated for another iteration. Figure 3C shows the assessment stage of the iteration. The requirements imposed in this stage are consistent with the established factor analysis guidelines [46].

#### Confirmatory Factor Analysis Summary

Next, the fit of the hypothesized model was confirmed or rejected by applying confirmatory factor analysis (CFA) using the other half of the data from 2011 to 2015. The goal of CFA is to confirm or reject the hypothesized model. As a result, (1) only observed variables were included, (2) the variables were loaded onto the same factors as in the CFA, and (3) the communality of the variables in the model was assessed. The model was confirmed if it satisfied the same requirements as specified for EFA [46].

#### Factor Restrictions and Limitations

**Figure 4:**
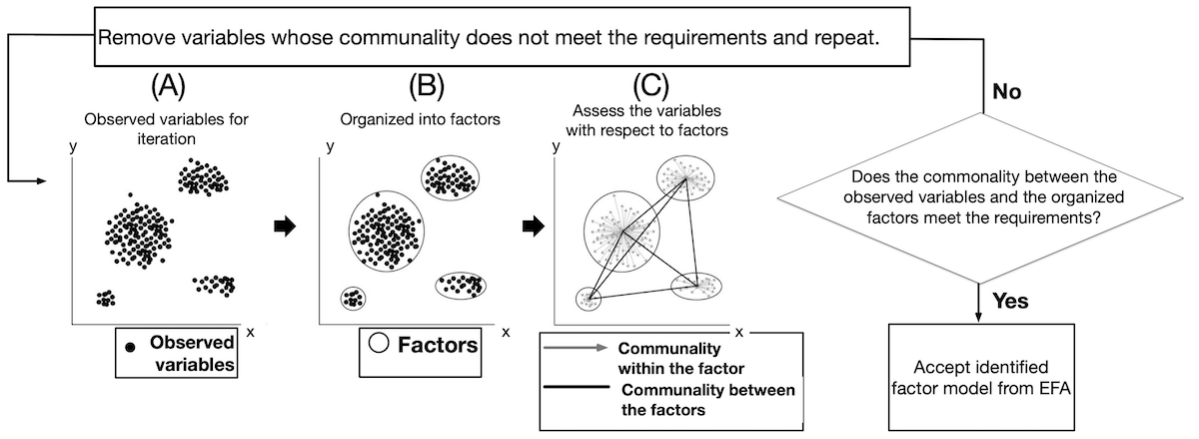
The three stages of an EFA iteration—(A) observed variable identification, (B) organization of variables into factors, and (C) assessment of the communality of variables within and between each of the identified factors. EFA: exploratory factor analysis.

The application of factor analysis imposes several limitations on our approach for estimating the health effects of adding bicycle and pedestrian paths to the city-specific factor model. First, a model that meets our requirements must be generated using EFA and confirmed using CFA. Furthermore, to apply our algorithm, the model must consist of at least three factors reflecting residents’ (1) DBC, (2) health, and (3) BPH. Finally, the health factor must include at least one observed variable related to a health outcome, and the BPH must include an observed variable related to the amount of bicycle and pedestrian path mileage in the census tract. The process of generating a factor model and determining whether it meets these restrictions is illustrated in Figure 4.

We imposed these restrictions because our health outcome prediction algorithm computes the factor scores for each census tract in a city based on these factors. Factor scores are continuous numbers reflecting the extent to which each census tract manifests each factor. For each factor, the scores were distributed normally, with a mean of 0 and an SD of 1. Large positive values reflect census tracts where the factor is heavily present, and large negative values reflect census tracts where the factor is not present at all [47].

Without these factors, the proposed algorithm could not be applied. It does not have sufficient data or structure to produce estimates of the health effects of adding bicycle and pedestrian paths. This is a limitation of the proposed approach. This limitation is discussed in more detail in the *Discussion* section.

### Estimating the Health Effects of Adding Bicycle Paths at the Census Tract Level

#### Overview

Given a factor model hypothesized by EFA and confirmed by CFA, we proposed an algorithm to predict the health effects of adding bicycle and pedestrian paths at the census tract level. For this purpose, we defined the input as an observed variable identified from the factor model. The variable then progressed through a sequence of steps that were applied to each census track and resulted in a predicted health outcome change for each identified health factor. The steps of this algorithm are enumerated in the following sections. Finally, the output from the algorithm was a list of hypothesized health improvement outcomes.

#### Input

In our problem statement, there was only one observed variable in the model that could be changed directly by a city-level planner, public health official, or decision maker. This variable represented the additional bicycle and pedestrian path mileage for a census tract within a city. This was the input to our algorithm, along with the factor model generated for the city.

#### Algorithm

**Figure 5:**
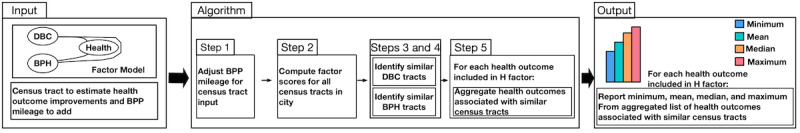
Instantiation of the algorithm for predicting how much additional BPP mileage in a census tract will improve health outcomes. BPH: bicycling and pedestrian habits; BPP: bicycle and pedestrian path; DBC: demographics and background characteristics.

The algorithm proceeded as follows, as conveyed visually in Figure 5.

1. The algorithm adds the bicycle and pedestrian path mileage to the specified census tract in the data set for the city.
2. Factor scores are computed for the following three factors: DBC, health, and BPH.
3. Given the DBC factor score for the input census tract, the algorithm identifies all other census tracts in the city with a DBC factor score within the threshold value—x. This list of census tracts reflects those that are similar to the input census tract with respect to the DBC factor. Recall that the factor scores are normally distributed, with an SD of 1. Thus, a census tract within a factor score x of the tract being analyzed reflects a census tract within SDs of the input tract [47].
4. Given the BPH factor score for the input census tract (which includes the newly added bicycle and pedestrian path mileage), the algorithm identifies all other census tracts in the city with BPH factor scores within x. This list of census tracts reflects those that are similar to the input census tract with respect to the BPH factor.
5. For each observed health outcome within the health factor, the algorithm creates a list that stores the difference between the value of the health outcome for each census tract identified in steps 3 and 4 and the value of the health outcome for the input census tract. This list of differences is a distribution of hypothesized improvements in a health outcome by adding a specified amount of bicycle and pedestrian path mileage to a census tract. Any differences that are <0 are discarded because these differences indicate that adding bicycle and pedestrian path mileage to the census tract will degrade health outcomes.

#### Output

For each list of hypothesized improvements for health outcomes generated in step 5, the algorithm output the minimum, mean, median, and maximum values of the improvements to the user. The algorithm could also report the entire distribution of possible improvements and SD of the distribution for each health outcome.

## Results

### Overview

The accuracy of our algorithm was elucidated through an empirical evaluation of alternative approaches for two different cities (Norfolk, Virginia, and San Francisco, California). In our evaluation, we computed how accurately each approach predicted the health outcome improvements of the bicycle and pedestrian paths added in each city in 2016. Specifically, for a given census tract, in each city that added bicycle and pedestrian paths miles in 2016, we evaluated how accurately our algorithm estimated an improvement in health outcomes in 2020. We chose to use a 5-year time-lapse period for our evaluation because research has shown that is the expected amount of time for a fully realized change in health outcomes given outdoor exercise infrastructure interventions [48,49].

### Factor Analyses

**Figure 6:**
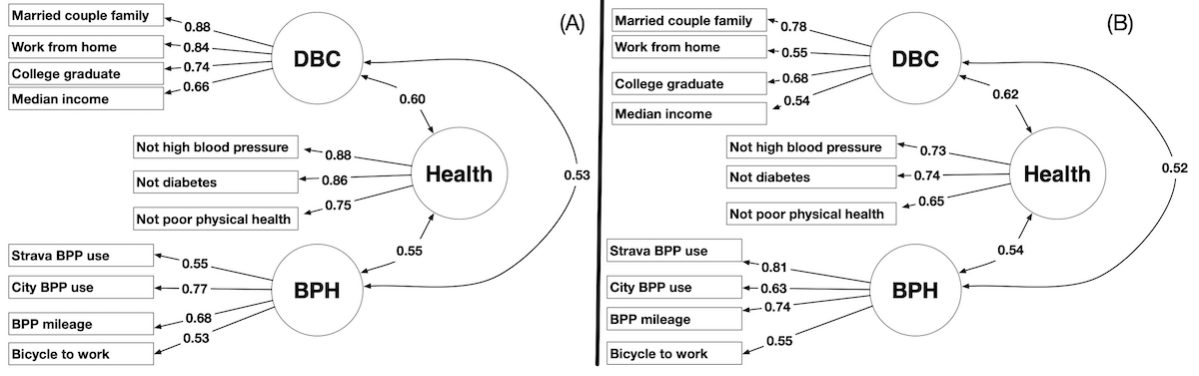
Exploratory factor analysis and confirmation factor analysis models for Norfolk, Virginia, using data sets from 2011 to 2015. Single-headed arrows reflect the commonality of an observed variable with a factor. Double-headed arrows reflect the value of the shared variance between factors. BPH: bicycling/pedestrian habits; BPP: bicycle and pedestrian path; DBC: demographics and background characteristics.

**Figure 7:**
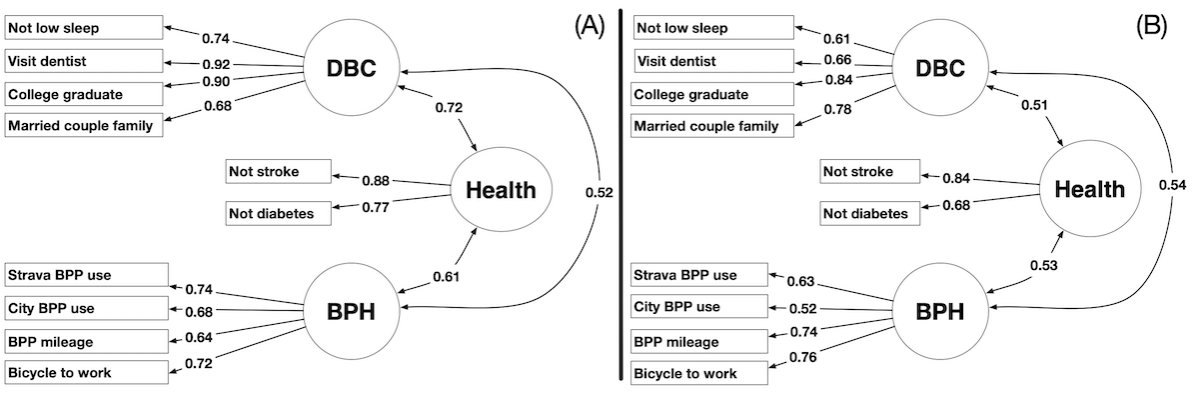
Exploratory factor analysis and confirmation factor analysis models for San Francisco, CA, using data sets from 2011 to 2015. Single-headed arrows reflect the commonality of an observed variable with a factor. Double-headed arrows reflect the value of the shared variance between factors. BPH: bicycling and pedestrian habits; BPP: bicycle and pedestrian path; DBC: demographics and background characteristics.

Applying the process described in the *Methods* section and shown in Figures 3 and 4 with the data from half the census tracts in each city for each year from 2011 to 2015 yields the EFA models shown in Figure 6A (n=195) and Figure 7A (n=490). Confirmation of these models using the remaining half of the census in each city for each year from 2011 to 2015 is shown in Figure 6B (n=190) and Figure 7B (n=485). Within the figures, the numbers labeled with single-headed arrows reflect the commonality of an observed variable with the associated factor. The double-headed arrows reflect the shared variance between factors [44,46]. The goodness-of-fit statistics corresponding to the CFA for each model are provided in Multimedia Appendix 6 (Norfolk, Virginia) and Multimedia Appendix 6 (San Francisco, California) along with guidelines on how to interpret the goodness-of-fit statistics.

Figures 6 and 7 show that the factor models for each city met our requirements. These models served as inputs for our estimation algorithm in the evaluation. It is important to note that although each model had the three required factors (DBC, health, and BPH), there were differences in the observed variables that form the factors. The factor analysis showed that changes in high blood pressure, diabetes, and poor physical health were predicted by changes in DBC and BPH in Norfolk, Virginia, whereas changes in stroke and diabetes were predicted by changes in DBC and BPH in San Francisco, California. This was not unexpected or a violation of the requirements of our approach. Although we required the 3 factors to be present, we anticipated that different observed variables would form these 3 factors for different cities.

### Evaluation

Recall that our algorithm took an input: (1) the factor model for a given city and (2) the census tract and amount of bicycle and pedestrian path mileage to be added. It then output the minimum, mean, median, and maximum estimated improvements by adding the bicycle and pedestrian path mileage to the input census tract. In the evaluation, we only used the median improvement estimate from the algorithm.

In our evaluation, we used our factor model constructed using data from 2011 to 2015 to estimate the accuracy of our approach and 2 alternative approaches with respect to the improvements in health outcomes provided by bicycle and pedestrian paths installed in 2016. The evaluation included 31.58 miles (50.81 km) of bicycle and pedestrian paths added in Norfolk, Virginia, across 31 census tracts and 52.36 miles (84.25 km) of bicycle and pedestrian paths added tracts in San Francisco, California, across 49 census tracts. Table 1 provides additional details regarding the setup of the evaluation.

**Table 1:** Evaluation setup metadata for Norfolk, Virginia, and San Francisco, California, in 2016.

	Norfolk, Virginia	San Francisco, California
BPP^a^ miles (km) added	31.58 (50.81)	52.36 (84.25)
Census tracts with paths added, n	31	49
Census tracts in city, n	77	195
Health outcomes evaluated	Diabetes %; poor physical health %; high blood pressure %	Diabetes %; stroke %

^a^BPP: bicycle and pedestrian path.

### Alternative Approaches

We evaluated our algorithm using 2 alternative approaches. The first alternative assumed that each health outcome within a census tract in the future would be same as the average value for that health outcome for the census tract from 2011 to 2015. This approach mirrored the prediction that the temperature tomorrow would be the same as the average temperature of the previous 5 days.

The second alternative used linear regression modeling [50]. This approach used regression to predict future changes in each health outcome using a weighted linear combination of the (1) DBC factor and (2) BPH factor scores of the census tract based on the constructed factor model using data from 2011 to 2015, after the specified increase in mileage.

### Approach

We evaluated our approach by using x=0.50. Recall that x is the threshold used to identify similar census tracts in terms of the (1) DBC factor and (2) BPH factor scores. In addition, our evaluation approach is an extension of the algorithm described in the *Methods* section. For our evaluation, given a specified number of bicycle and pedestrian path miles to be added and a census tract, we ran the algorithm for every 0.10-mile increment of bicycle and pedestrian paths up to the specified number of miles.

**Figure 8:**
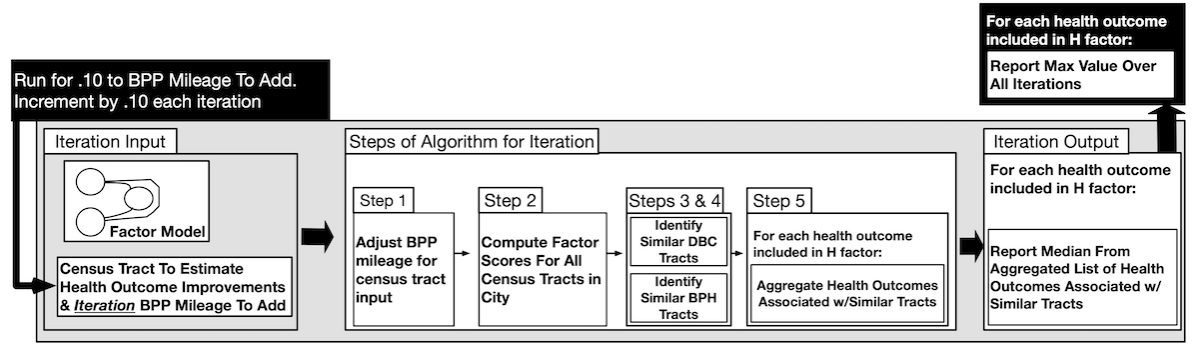
The specific version of our algorithm included in the applied evaluation. BPP: bicycle and pedestrian path; BPH: bicycling and pedestrian habits; DBC: demographics and background characteristics.

Each time the algorithm was executed, the median improvement from the algorithm was collected. The largest improvement over all the runs was reported. A version of our approach is shown in Figure 8. It implemented the assumption that adding more bicycle and pedestrian path mileage (ie, 1.0 miles as opposed to 0.5 miles) to a given census tract would not be detrimental to the expected improvement in a health outcome.

### Measures of Effectiveness

For a given city and a given approach to estimating the improvement in a health outcome for bicycle and pedestrian paths added in 2016, we computed the following two measures of effectiveness (MOEs): (1) the root mean squared error (RMSE) and (2) the mean absolute error (MAE). These are 2 established metrics used to measure the accuracy of continuous variables. MAE measures the average magnitude of the errors in a set of predictions without considering their direction. It reflects the average over the evaluation of the absolute differences between the prediction and actual observation where all individual differences have equal weight. RMSE also measures the average magnitude of the error. However, it reflects the square root of the average squared differences between the predicted and actual observations. Within the RMSE, the errors were squared before they were averaged. As a result, the RMSE gives a relatively high weight to large errors [51]. By using both metrics as MOEs, we could capture the accuracy of each approach for decision makers who (1) view all errors equally (MAE) and (2) view large errors as particularly undesirable (RMSE).

### Measures of Success

We deem our approach successful if, for each city included in our evaluation, our approach is more accurate across every MOE than the best alternative approach, and these differences are all statistically significant at *P*<.01, when a 1-tailed paired sample *t* test is applied. We used a 1-tailed paired sample *t* test to determine whether the mean paired difference between the MOEs of our approach and an alternate approach was <0 (ie, our approach was more accurate). In this procedure, paired observations reflected the MOEs for a given census tract. Within the pair, one observation corresponded to our approach, and the other corresponded to an alternative approach [52].

## Discussion

### Principal Findings

In our evaluation, we compare the accuracy of our factor model approach, a linear regression approach, and predict no change approach. Each approach estimates the improvements in health outcomes provided by bicycle and pedestrian paths installed in 2016 in 31 census tracts in Norfolk, Virginia and 49 census tracts in San Francisco, California. The results of the evaluation are shown in Table 2.

Table 3 shows that our approach is more accurate than the alternatives, and Table 4 shows that those improvements in accuracy over the best alternative are statistically significant because *P*<.001 for every health outcome in each city when the 1-tailed paired *t* test is applied.

We expected our approach to outperform the “predict no change approach” because the CDC 500 Cities project and bicycle and pedestrian path data for both cities show that most of the time when a bicycle path of any length is added, the health outcomes identified by the factor analysis improve within 5 years. However, we did not know whether our approach outperformed the linear regression approach.

The results of the evaluation showed that our approach outperformed the linear regression models because it assumed that critical thresholds within the DBC and BPH factors existed (parameter x in steps 3 and 4 of the algorithm). The linear regression approach did not make this assumption [50]. By accounting for this threshold, our approach ensured that it did not overpredict the improvement offered by additional bicycle path miles when the DBC or BPH factor for the census tract indicated that the additional path miles would be ineffective.

By not accounting for this threshold, the linear regression approach could overpredict the expected improvement in health outcomes within a census tract. This was because the linear regression approach assumed that some amount of bicycle and pedestrian paths in each census tract would yield a population without any negative health outcomes. This is unrealistic. Our evaluation results in Tables 3 and 4 demonstrate that linear regression yields statistically significant inferior accuracy, as measured by our 1-tailed paired *t* test.

**Table 2:** Evaluation of approaches for bicycle and pedestrian paths added in Norfolk, Virginia, in 2016.

Health outcome and MOE^a^ (% of individuals who experience a negative health outcome)		Predict no change (census tract: n=31), mean (SD)	Linear regression (census tract: n=31), mean (SD)	Our approach (census tract: n=31), mean (SD)
**Diabetes**				
	MAE^b^	2.33 (0.66)	2.14 (0.67)	1.63 (0.59)
	RMSE^c^	2.41 (0.62)	2.29 (0.61)	1.67 (0.55)
**Poor physical health**				
	MAE	2.69 (0.72)	2.21 (0.69)	1.83 (0.57)
	RMSE	2.64 (0.69)	2.27 (0.66)	1.94 (0.56)
**High blood pressure**				
	MAE	2.95 (1.17)	2.27 (1.07)	1.49 (0.85)
	RMSE	3.18 (1.13)	2.38 (0.92)	1.55 (0.82)

^a^MOE: measure of effectiveness.

^b^MAE: mean absolute error.

^c^RMSE: root mean squared error.

**Table 3:** Evaluation of approaches for bicycle and pedestrian paths added in San Francisco, California, in 2016.

Health outcome and MOE^a^ (% of individuals who experience a negative health outcome)		Predict no change (census tract: n=49), mean (SD)	Linear regression (census tract: n=49), mean (SD)	Our approach (census tract: n=49), mean (SD)
**Diabetes**				
	MAE^b^	2.32 (1.19)	2.18 (1.18)	1.24 (0.91)
	RMSE^c^	2.44 (1.11)	2.41 (1.11)	1.35 (0.90)
**Stroke**				
	MAE	2.68 (0.58)	2.78 (0.68)	1.81 (0.52)
	RMSE	3.19 (0.52)	2.97 (0.64)	1.88 (0.49)

^a^MOE: measure of effectiveness.

^b^MAE: mean absolute error.

^c^RMSE: root mean squared error.

**Table 4:** Assessment of whether the improved accuracy of bicycle and pedestrian paths added in 2016 is statistically significant.

City, health outcome, and MOE^a^			Statistical significance of our approach MOE versus best alternative MOE, *P* value
**Norfolk, Virginia (census tract: n=31)**			
	**Diabetes**		
		MAE^b^	<.001
		RMSE^c^	<.001
	**Poor physical health**		
		MAE	<.001
		RMSE	<.001
	**High blood pressure**		
		MAE	<.001
		RMSE	<.001
**San Francisco, California (census tract: n=49)**			
	**Diabetes**		
		MAE	<.001
		RMSE	<.001
	**Stroke**		
		MAE	<.001
		RMSE	<.001

^a^MOE: measure of effectiveness.

^b^MAE: mean absolute error.

^c^RMSE: root mean squared error.

### Comparison With Prior Work

Our study builds on a significant amount of previous research. Numerous researchers have used statistical analyses to (1) explore the health effects of commuting via bicycle or by foot [4,53-62] and (2) assess the health benefits of bicycling and bicycle and pedestrian paths versus the risk of injury or death [63-67]. This study captured data related to walking and bicycling using telephone and web-based surveys [53,54,68], GPS, accelerometers, heart rate monitors [6,58,69-77], bicycling shares [78-80], and social media [17,81].

Predicting which bicycle and pedestrian paths residents will choose is also related to our work. Within this arena, researchers have found different results with respect to the extent to which bicycle and pedestrian path users prefer to take paths that minimize the total travel distance. For example, Broach et al [71,82] used data from Portland, Oregon, to formulate a model that estimated that preferred routes were <10% longer than the shortest path distance. Similarly, Winters et al [39] found that 75% of trips in Vancouver, British Columbia, Canada, were within 10% of the shortest path distance. However, Aultman-Hall et al [83] found no clear relationship between the shortest path distance and percent route deviation in Ontario, Canada, and Krizek et al [84] looked at data in Minneapolis, Minnesota, and found that the average path traveled was roughly twice as long as the shortest path available.

There is also significant research focused on understanding the rate at which future use of bicycle and pedestrian paths will change, as commuters who currently do not use bicycle and pedestrian paths start to transition into commuting by foot or bicycle. Waldykowski et al [85] developed a simulation that explored the conditions under which motor vehicle commuters switch over to commute by bicycle and pedestrian path [85]. Similarly, Mahfouz et al [86] combined distance decay, route calculation, and network analysis methods to examine (1) where future bicycle and pedestrian path commuter demand is within a city, (2) if it is likely to rise, and (3) how such demand could be accommodated within existing bicycle and pedestrian path networks. Finally, Liu et al [87] proposed a connectivity measure that captures the importance of a link in connecting the origins of cyclists and nearby subway stations and incorporated it into a statistical model.

In addition, researchers have attempted to better understand the impact of bicycle and pedestrian paths on health outcomes. This work includes (1) cost-benefit analysis of bicycle and pedestrian paths with respect to health improvements [10,88]; (2) lessons learned from cities with especially enthusiastic cycling culture such as Amsterdam, the Netherlands; Barcelona, Spain; and Chicago, Illinois [49,89,90]; and (3) understanding what type of bicycle and pedestrian paths cyclists and pedestrians prefer [69].

These studies demonstrate the need for granular analysis with actionable outcomes with respect to bicycle and pedestrian paths. Furthermore, although the studies have had a significant impact on the research community, none of them constructed a city-specific model to advise decision makers about the extent to which adding bicycle and pedestrian paths to a census tract would improve residents’ health outcomes. Our study addresses this problem within a larger bicycle and pedestrian path research area.

### Limitations

#### Data Limitations

Strava has emerged as a tool of interest for collecting data on bicycling, running, and walking, understanding the effects of new interventions for users, and promoting safety among riders. However, this crowdsourced data are biased toward recreational riders, who are frequent users of GPS-enabled fitness apps. Thus, there is a need to quantify and correct the inherent bias in crowdsourced data to better represent all residents across various demographics. Strava users tend to be more frequently identified as male, be older, and have more income than the general population [17]. In addition, there are limitations to how well the data counted by municipalities reflect the actual volume of bicycle and pedestrian traffic on bicycle and pedestrian paths [91,92]. Research has shown that accounting for biases in placement, time, and day of the week needs to be performed to address these issues [93,94].

Controlling for these biases in the Strava and municipal count data is beyond the scope of our work. However, it is important to note that there were biases in the data. Ultimately, these limitations mean that the Strava data sets that informed our study are nonuniform subsamples of the traffic of cyclists, walkers, and runners in Norfolk, Virginia, and San Francisco, California.

It is also important to note that the use of e-bikes has changed significantly during the period of our study [6]. e-Bikes present a potential opportunity to encourage active transportation while reducing personal barriers to active transportation [95,96]. Survey results suggest that e-bikes may reduce some personal barriers to traditional cycling and allow riders to travel greater distances [97,98]. In addition, e-bikes may have the added benefit of promoting health among individuals who are otherwise reluctant to engage in physical activity [99] and improve metabolic fitness [100] and enjoyment [101]. Exploring how the increased use of e-bikes affects our approach is an opportunity for future work.

#### Approach Limitations

Recall that our approach uses 5 years of past data to fit a factor model and requires the factor model to consist of at least three factors where unique factors reflect residents’ (1) DBC, (2) health, and (3) BPH. In addition, the health factor must include at least one observed variable related to a health outcome, and the BPH factor must include an observed variable related to the amount of bicycle and pedestrian path mileage in the census tract. For cities in which these requirements cannot be met, our approach cannot be applied. This limits its utility and geographic area of applicability. However, related research has shown that these factors are important to account for and often present when understanding who chooses to use bicycle and pedestrian path and how effective bicycle and pedestrian paths are in improving health outcomes [2,56,78,102-104]. Furthermore, these factors provide a structure that enables our approach to predict improvements in health outcomes more accurately than the alternative approaches.

### Validity Threats

Threats to internal and external validity affected our study. Threats to internal validity arose when factors affected the dependent variables without evaluators’ knowledge. It is possible that some flaws in the implementation of our model could have affected the evaluation results. However, our approach used established libraries to conduct factor analysis, and the source code passed internal reviews [105,106].

Threats to external validity occur when evaluation results cannot be generalized. Although the evaluation was performed using more than 83 miles of added bicycle paths in 80 census tracts across the 2 cities, the factor models and accuracy results cannot necessarily be generalized to other areas. In addition, the factor analysis that generates our models assumes that each pair of variables follows a bivariate normal distribution. Although we verified that this assumption was true in our data, it may not be generalizable to other data sets and other cities where the approach is applied. However, it is very important to note that our approach, which yielded models producing these results, can be applied to other cities assuming that factor models that meet our requirements exist [105,106].

### Conclusions

Our work is directly actionable for policy makers, public health professionals, and urban planners in Norfolk, Virginia, and San Francisco, California, by providing concrete insight into the question “To what extent will adding specified bicycle and pedestrian path mileage to a census tract improve residents’ health outcomes over time?” Specifically, it enables them to (1) weigh the extent to which 2 bicycle and pedestrian paths of equal cost proposed in 2 different census tracts improve the health outcomes of the residents, (2) identify areas where bicycle and pedestrian paths are unlikely to be effective public health interventions and other strategies should be used to help residents, and (3) quantify the minimum amount of bicycle path miles that need to be added in a given census tract to maximize the improvement in health outcomes for residents. Our results demonstrate that for 2 different cities, our approach estimates improvements in health outcomes more accurately than alternate approaches, and these improvements are statistically significant.

A web application that implements our algorithm and summarizes its findings in an actionable manner is available [107]. Multimedia Appendix 7 provides the source code for the web application. This application was used to identify a recommended set of bicycle and pedestrian paths across census tracts in Norfolk, Virginia. A time series forecast of the expected improvements in health outcomes for these recommendations was also conducted. These artifacts, which are examples of the types of analyses enabled by our approach, are available in Multimedia Appendix 8. A similar web application that implements our algorithm for San Francisco, California, is available [108]. The source code for it is provided in Multimedia Appendix 9.

## Appendix

Multimedia Appendix 1 American Community Survey data used for factor analysis.

Multimedia Appendix 2 The Centers for Disease Control and Prevention 500 Cities project data were used for factor analysis and evaluation.

Multimedia Appendix 3 City-supplied bicycle and pedestrian count and path length data used for factor analysis.

Multimedia Appendix 4 Strava-supplied bicycle and pedestrian trip count data used for factor analysis.

Multimedia Appendix 5 Prospective variables identified by subject matter experts for factor analysis.

Multimedia Appendix 6 Goodness-of-fit measure descriptions and statistics for generated factor models.

Multimedia Appendix 7 Data and source code for web application deployment of model for Norfolk, Virginia.

Multimedia Appendix 8 Data and source code for research artifacts produced from the model for Norfolk, Virginia, including recommended portfolios of bicycle and pedestrian paths and time series of expected health outcome improvements.

Multimedia Appendix 9 Data and source code for web application deployment of the model for San Francisco, California.

## Abbreviations

ACS: American Communities Survey
BPH: bicycling and pedestrian habits
CDC: Centers for Disease Control and Prevention
CFA: confirmatory factor analysis
DBC: demographics and background characteristics
EFA: exploratory factor analysis
MAE: mean absolute error
MOE: measure of effectiveness
RMSE: root mean squared error

## Data Availability

The contents of the multimedia appendices are specified below and are supplied in the paper. They are also available on the web as Mendeley Data [109].
