# Supplementary figures and images for: Estimating the Health Effects of Adding Bicycle and Pedestrian Paths at the Census Tract Level: Multiple Model Comparison
Source: JMIR Public Health Surveill. 2022 Aug 24;8(8):e37379. doi: 10.2196/37379 (PMC9453587; doi:10.2196/37379)

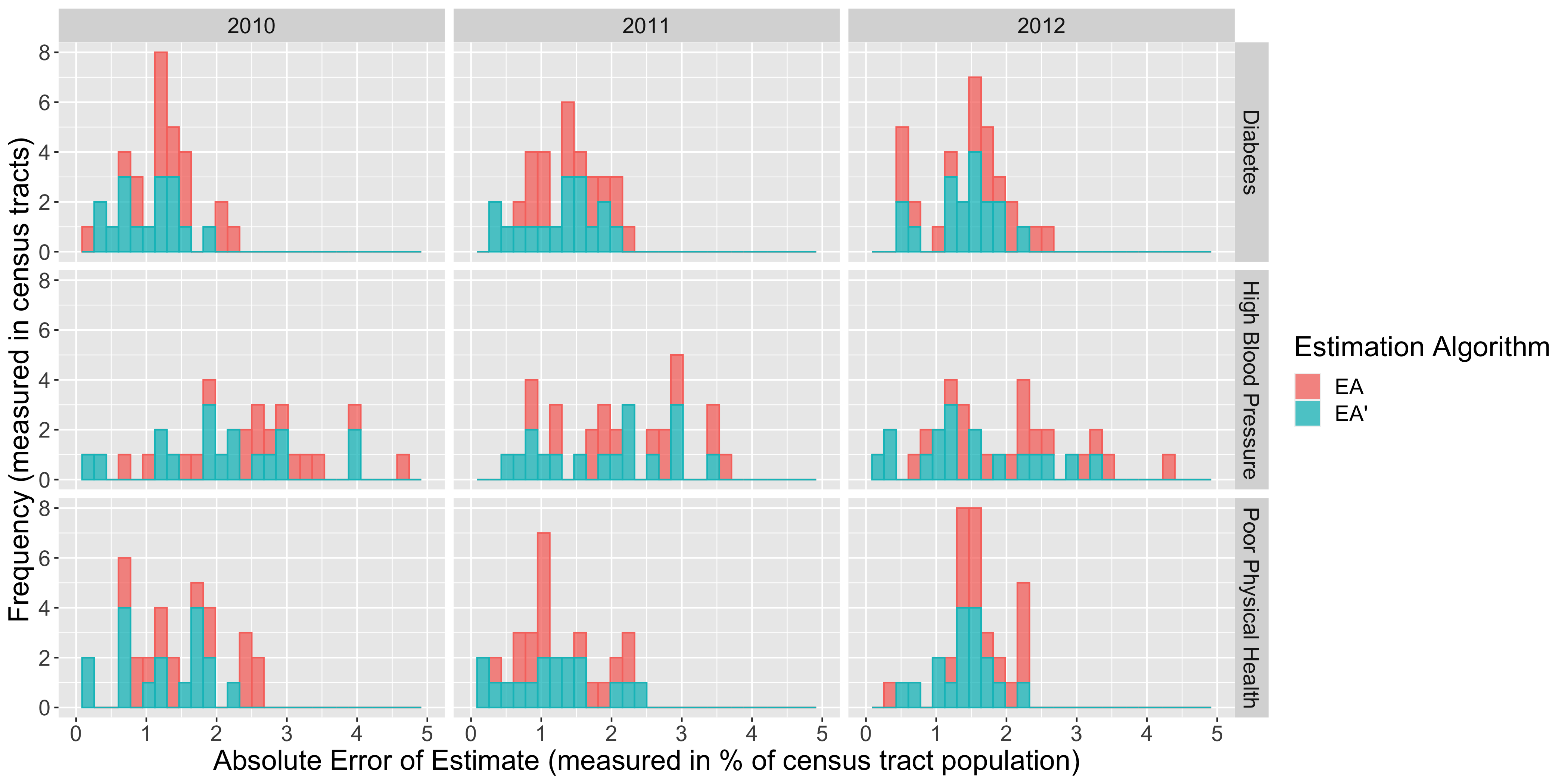

Supplement: Multimedia Appendix 7 [file publichealth_v8i8e37379_app7.zip › appendix_7/webapp/www/graphic_of_error_estimates.png]

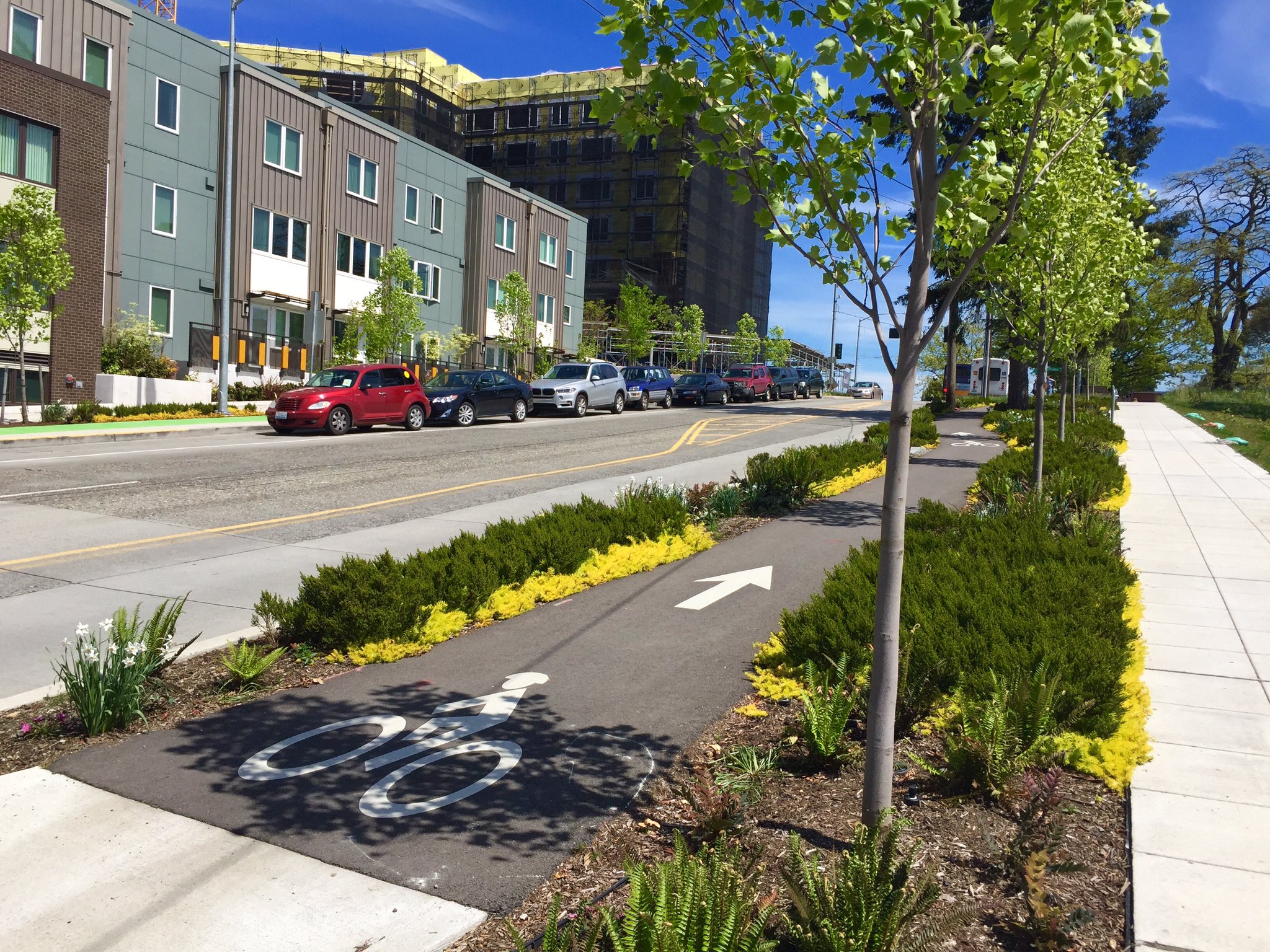

Supplement: Multimedia Appendix 7 [file publichealth_v8i8e37379_app7.zip › appendix_7/webapp/www/bike-path.jpeg]

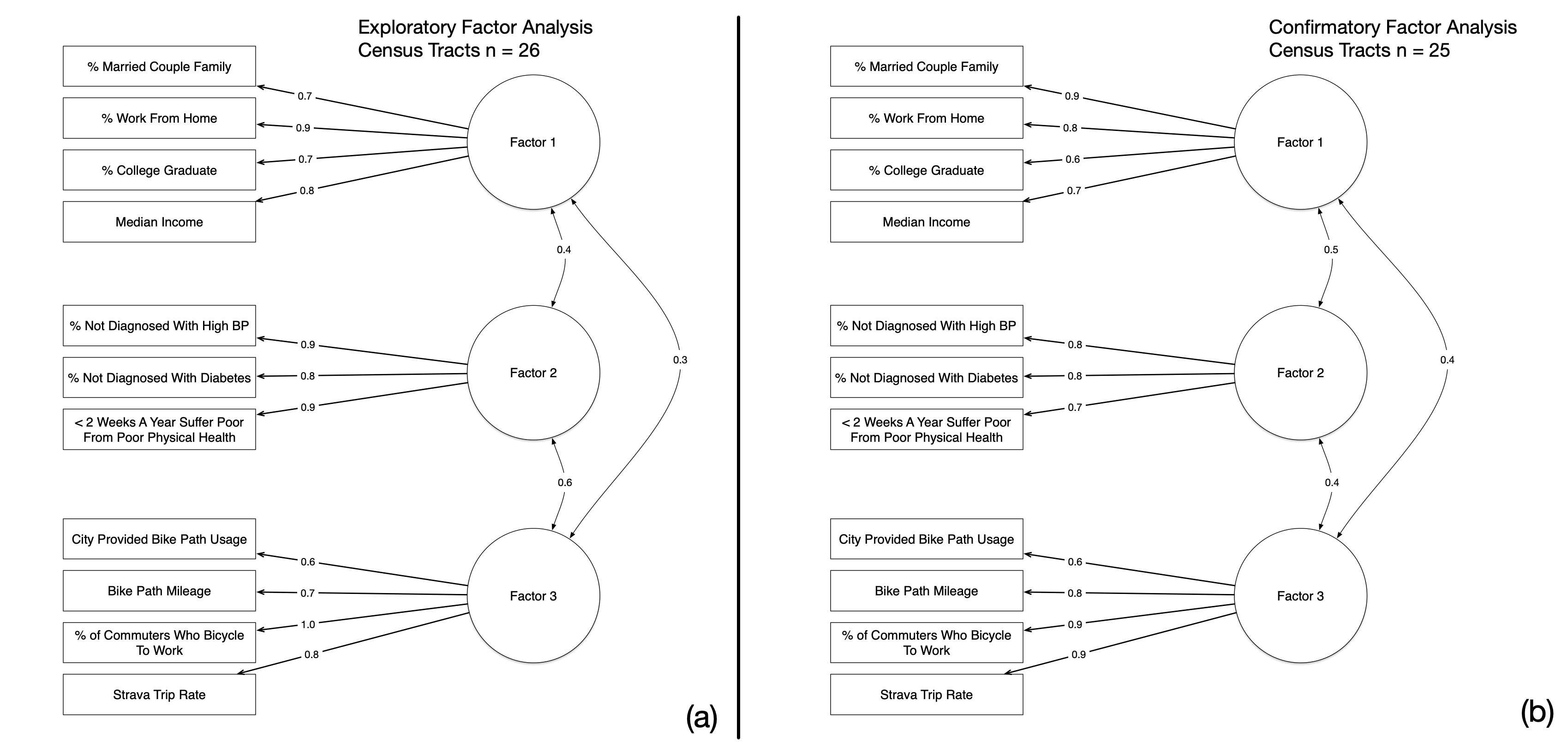

Supplement: Multimedia Appendix 7 [file publichealth_v8i8e37379_app7.zip › appendix_7/webapp/www/EFA-CFA.png]

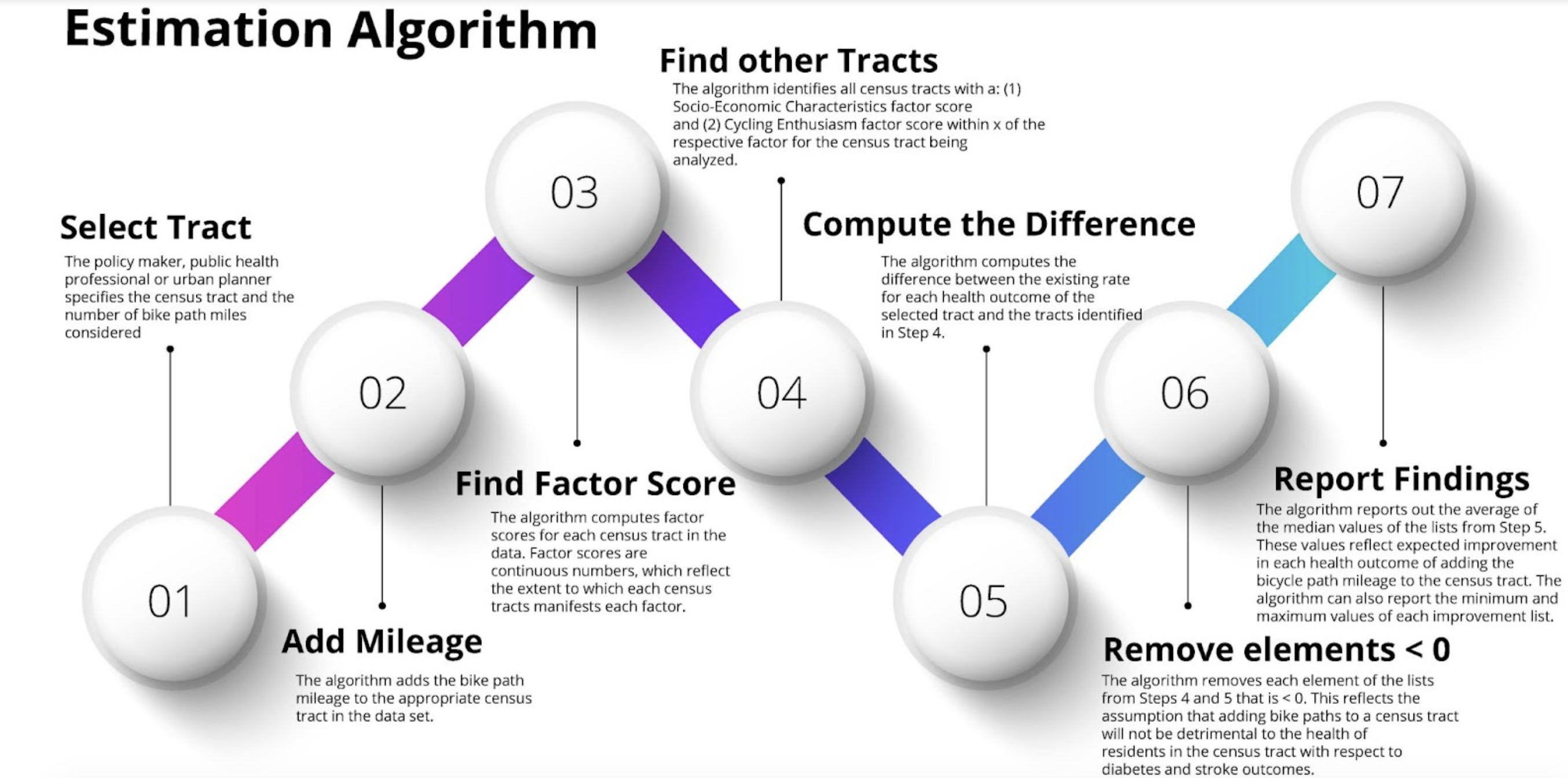

Supplement: Multimedia Appendix 7 [file publichealth_v8i8e37379_app7.zip › appendix_7/webapp/www/estimation-algorithm.png]

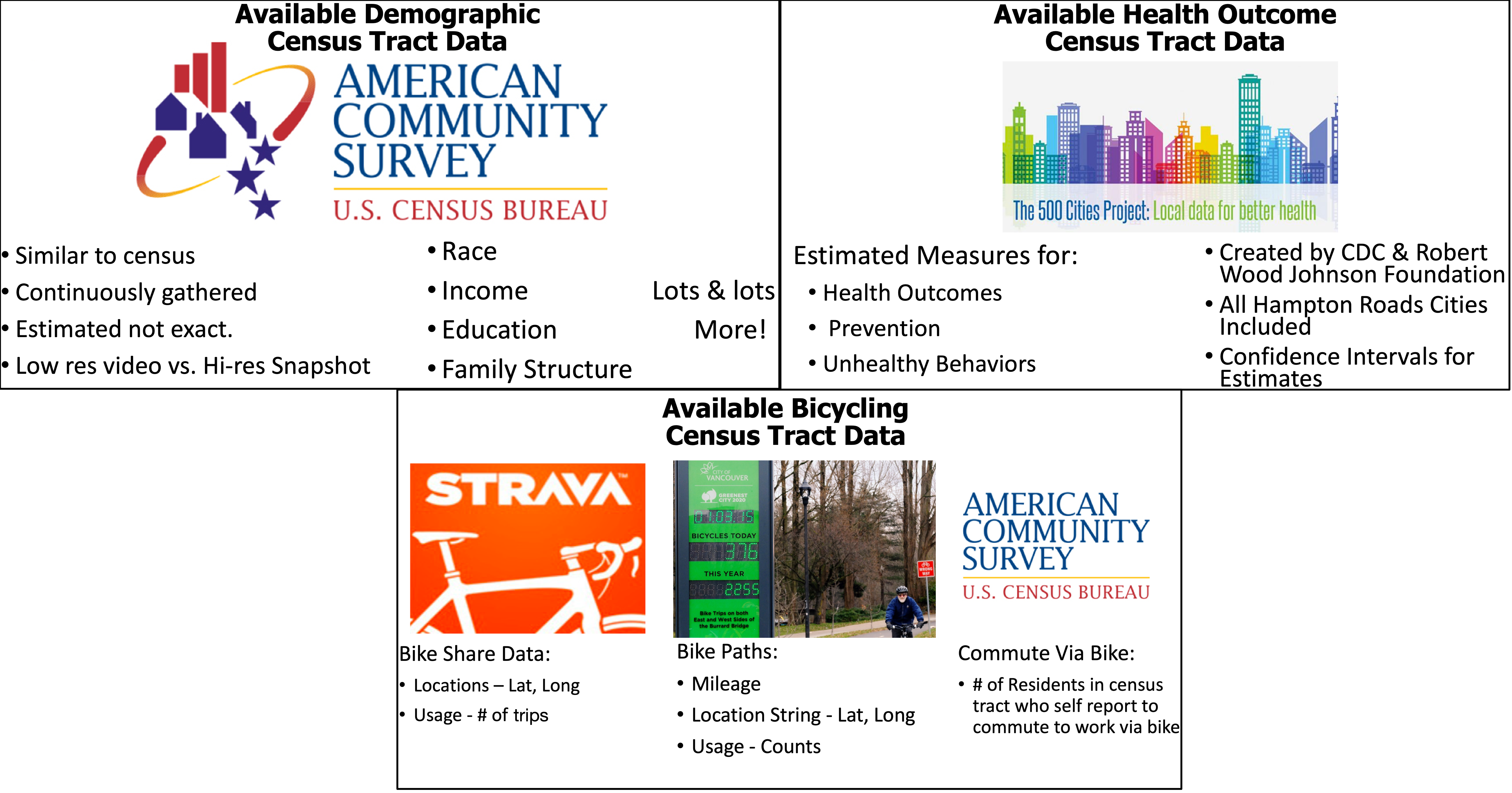

Supplement: Multimedia Appendix 7 [file publichealth_v8i8e37379_app7.zip › appendix_7/webapp/www/data.png]

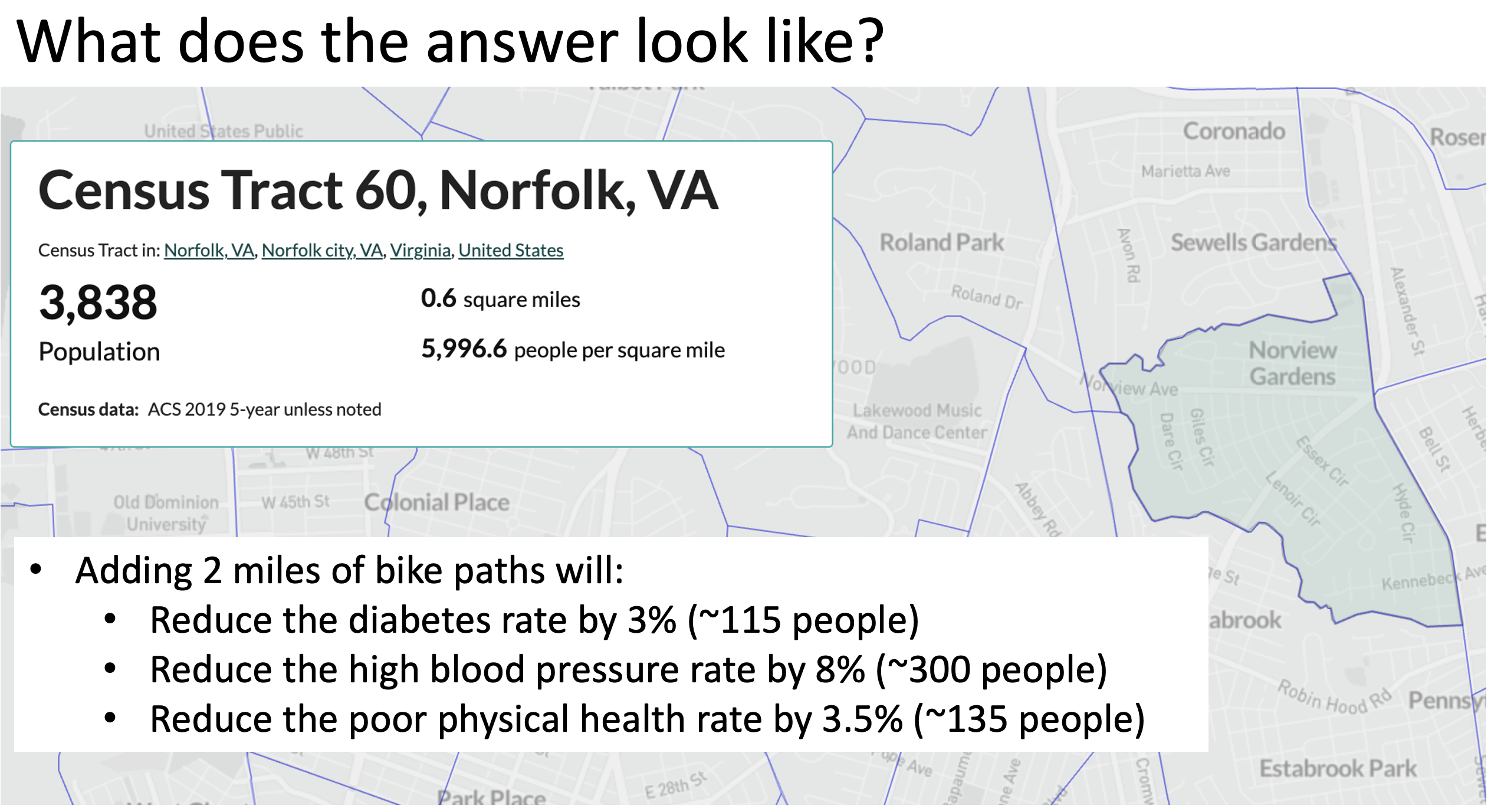

Supplement: Multimedia Appendix 7 [file publichealth_v8i8e37379_app7.zip › appendix_7/webapp/www/objective.png]

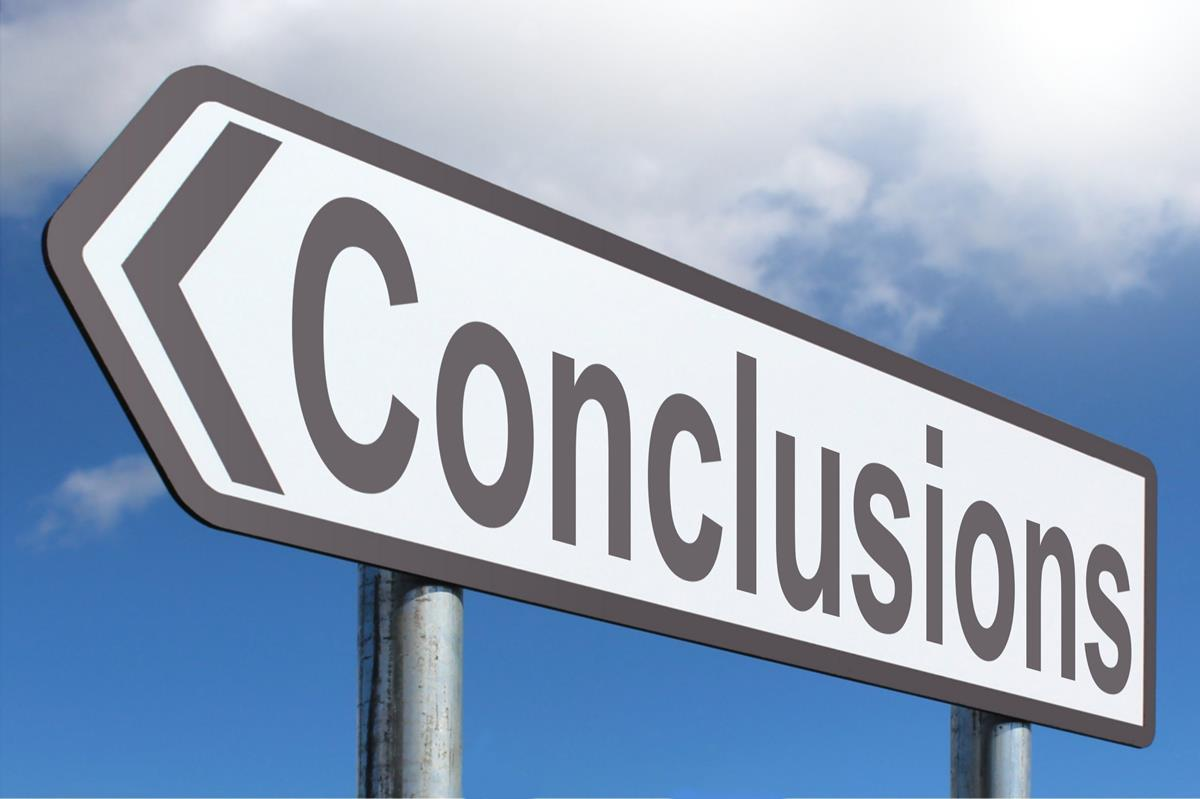

Supplement: Multimedia Appendix 7 [file publichealth_v8i8e37379_app7.zip › appendix_7/webapp/www/conclusions.png]

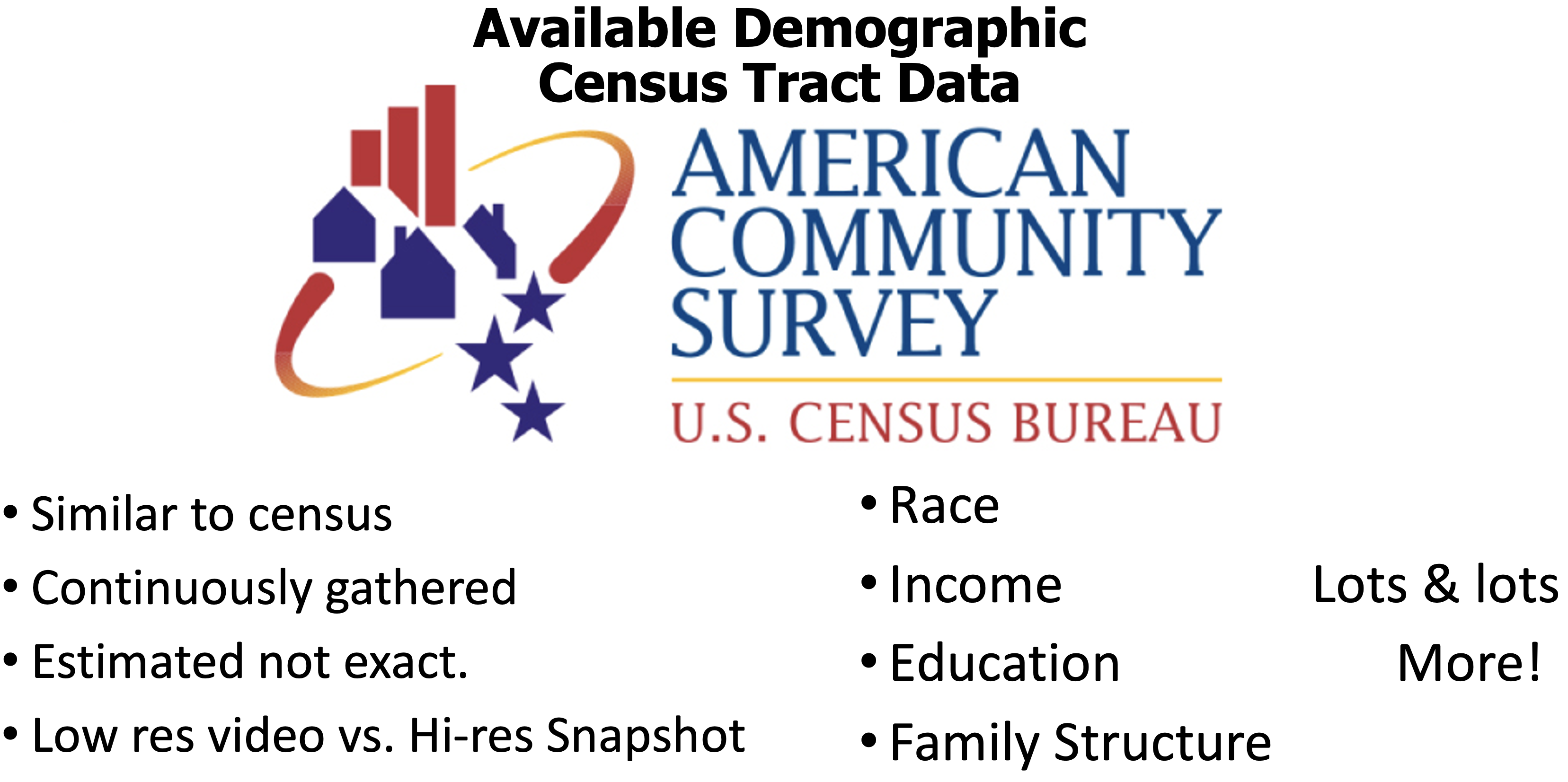

Supplement: Multimedia Appendix 7 [file publichealth_v8i8e37379_app7.zip › appendix_7/webapp/www/data.graffle/image1.tiff]

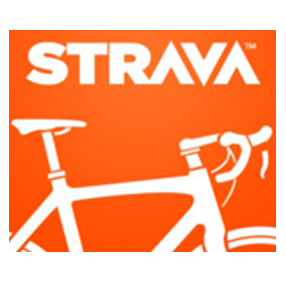

Supplement: Multimedia Appendix 7 [file publichealth_v8i8e37379_app7.zip › appendix_7/webapp/www/data.graffle/image6.tiff]

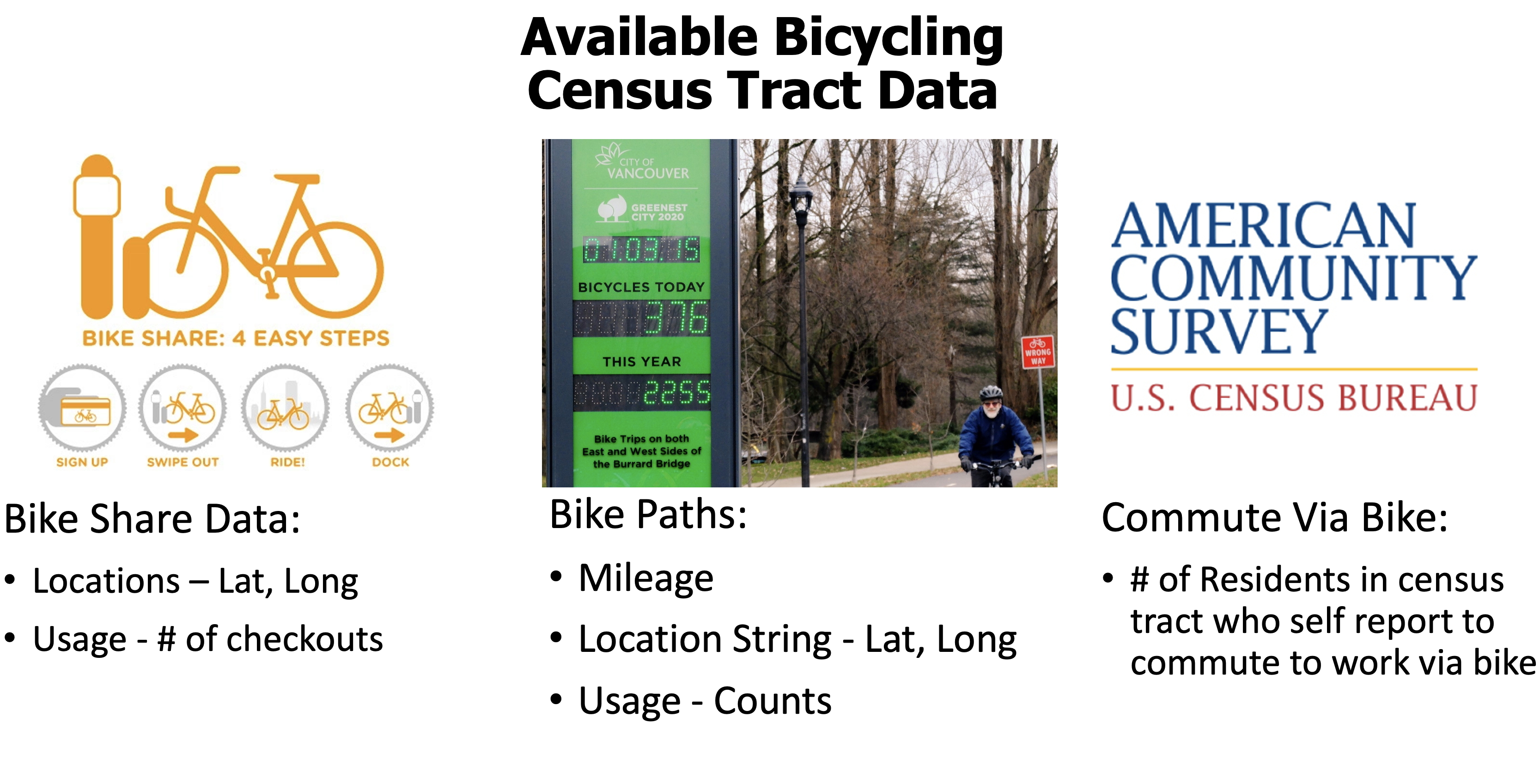

Supplement: Multimedia Appendix 7 [file publichealth_v8i8e37379_app7.zip › appendix_7/webapp/www/data.graffle/image4.tiff]

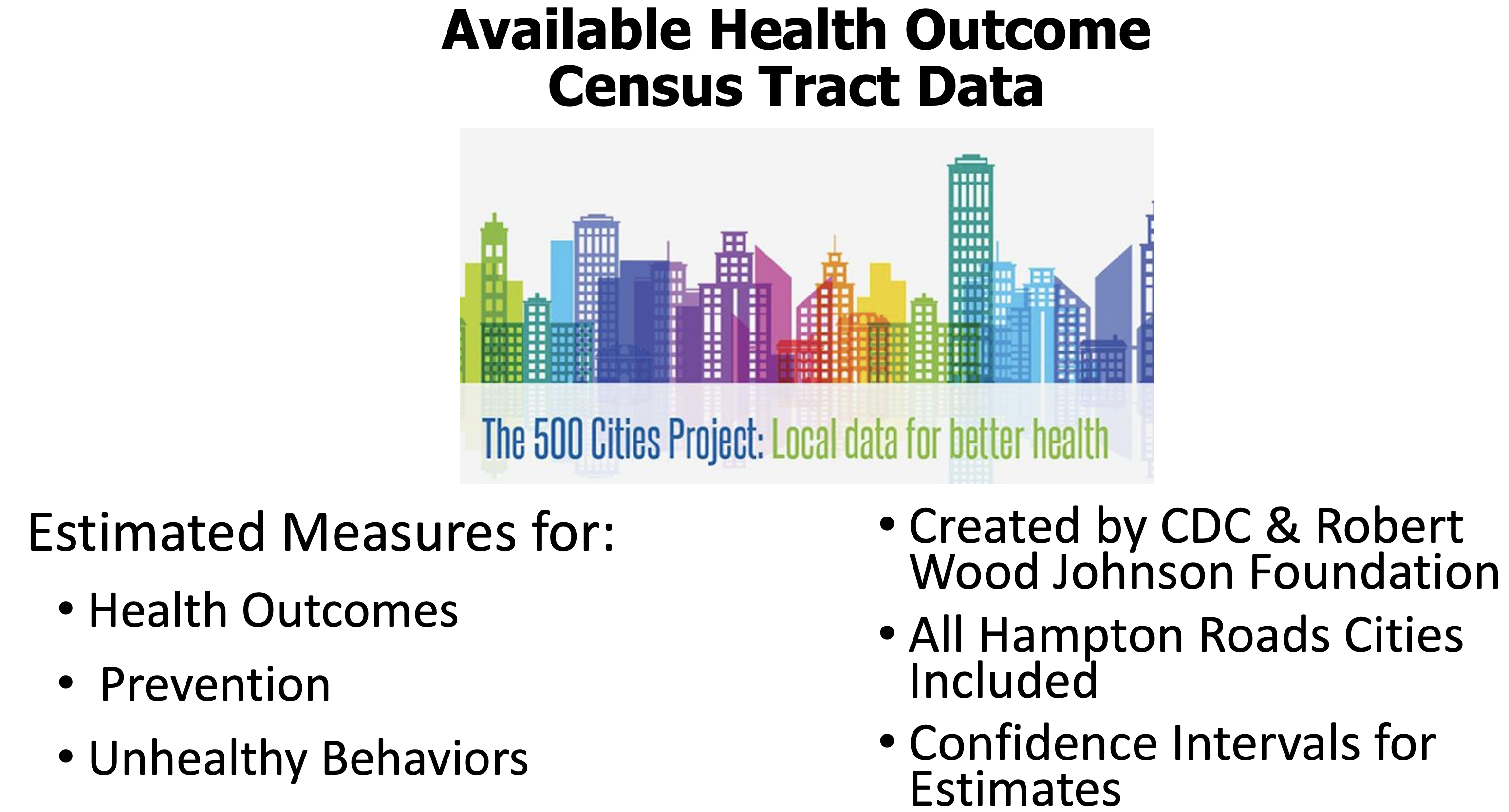

Supplement: Multimedia Appendix 7 [file publichealth_v8i8e37379_app7.zip › appendix_7/webapp/www/data.graffle/image3.tiff]

# Factor Analysis

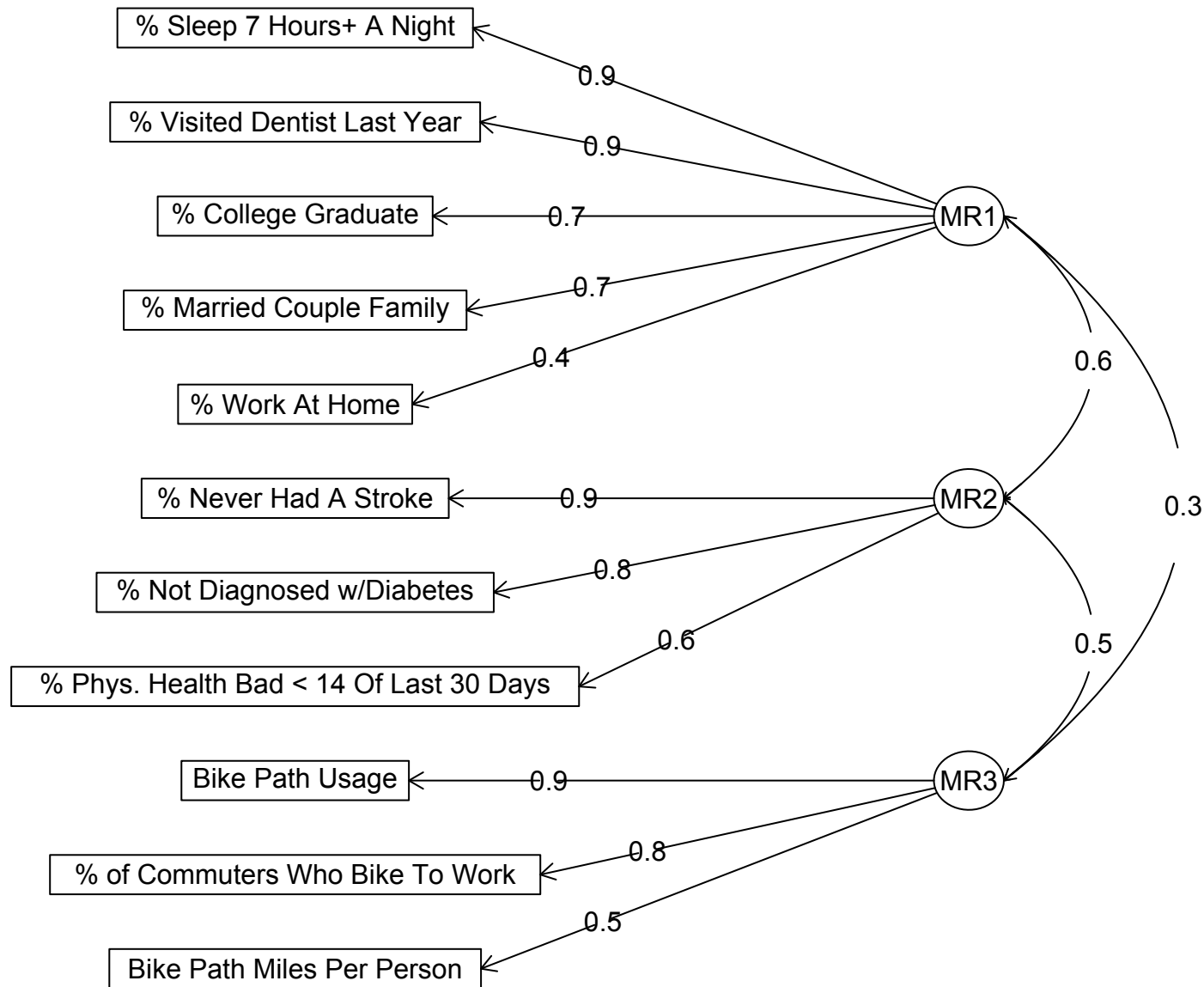

Supplement: Multimedia Appendix 9 [file publichealth_v8i8e37379_app9.zip › appendix_9/webapp/total-FactorAnalysisPlot.pdf]

# Exploratory Analysis n=98

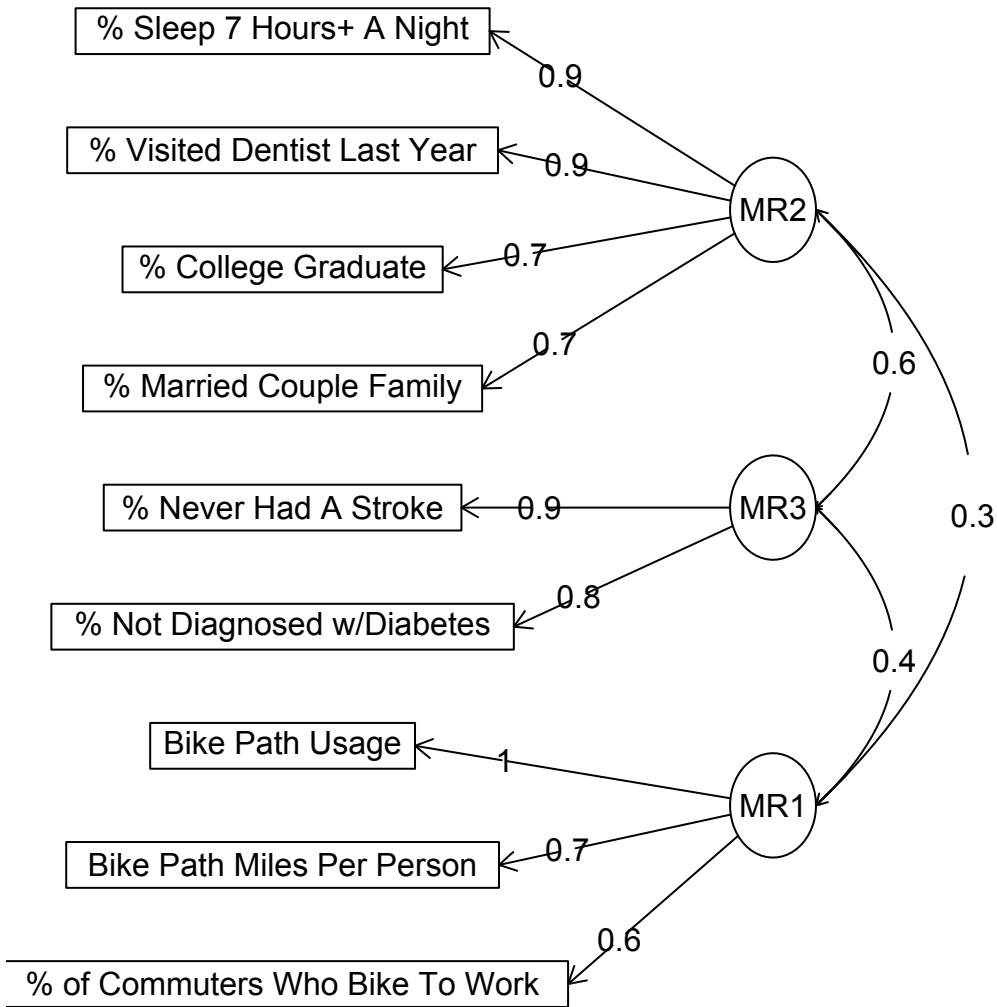

Supplement: Multimedia Appendix 9 [file publichealth_v8i8e37379_app9.zip › appendix_9/webapp/sf-exploratory-analysis-factor-plot.pdf]

# Projected Improvement In Health For Census Block 06075011100 If 20 Bike Path Miles Are Added

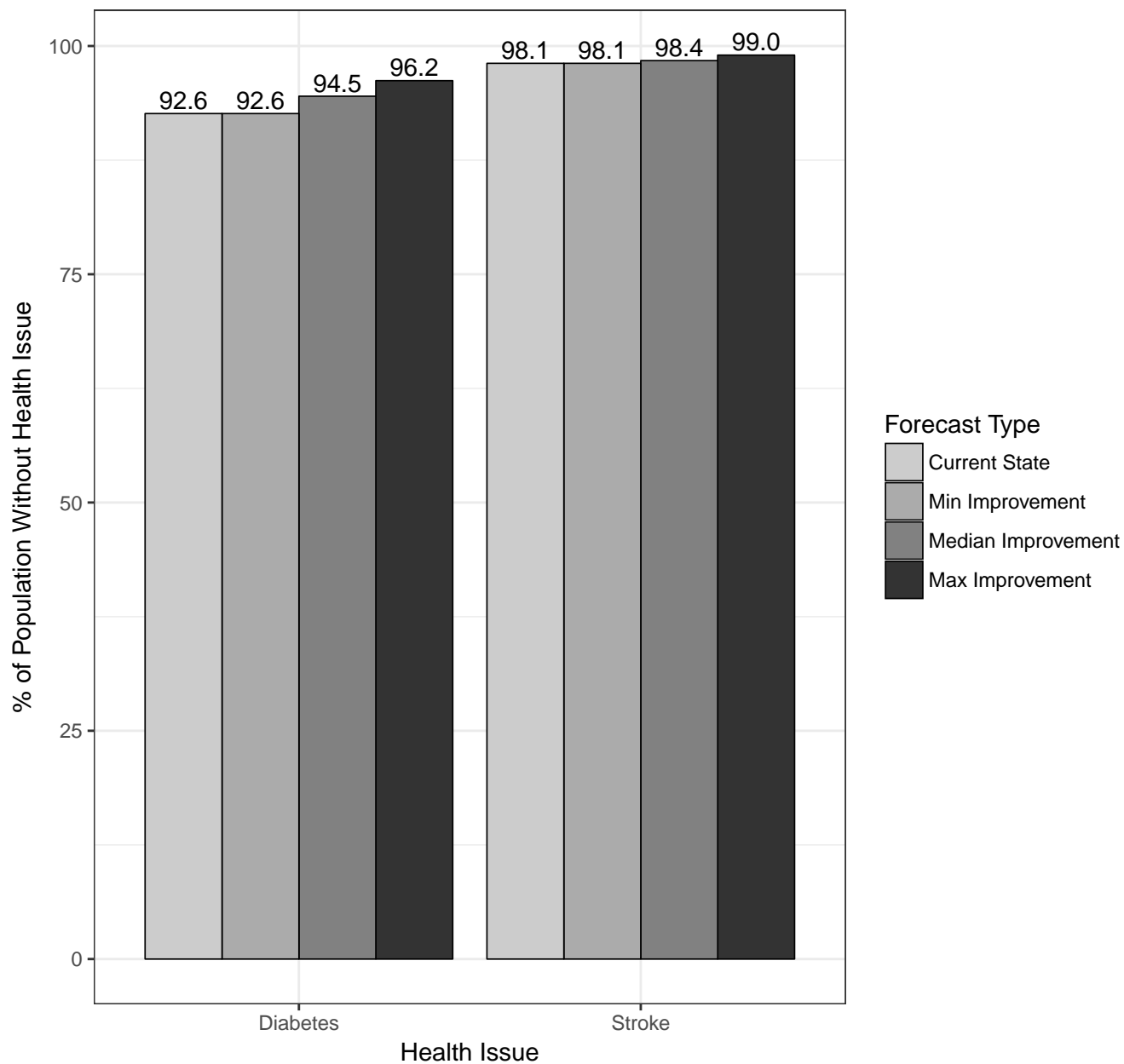

Supplement: Multimedia Appendix 9 [file publichealth_v8i8e37379_app9.zip › appendix_9/webapp/outputPlot.pdf]

# Confirmatory Analysis n=97

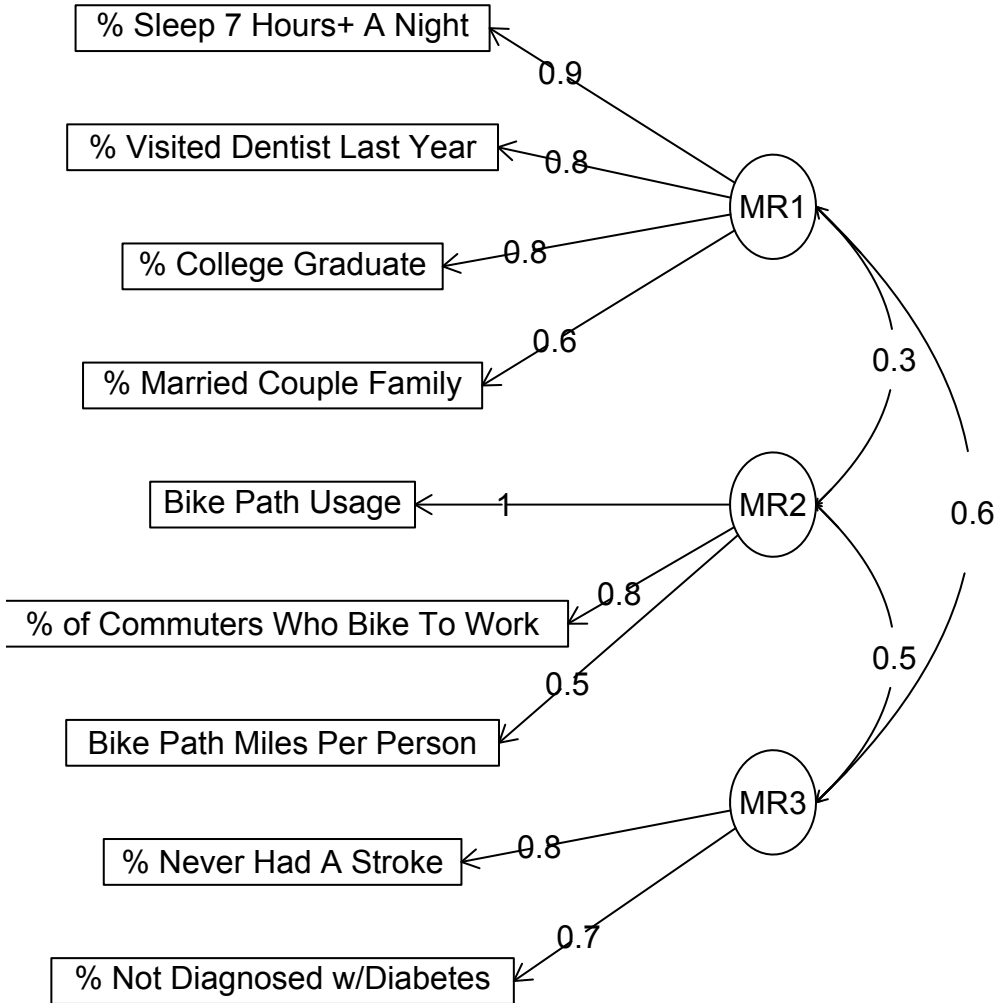

Supplement: Multimedia Appendix 9 [file publichealth_v8i8e37379_app9.zip › appendix_9/webapp/sf-confirmatory-analysis-factor-plot.pdf]

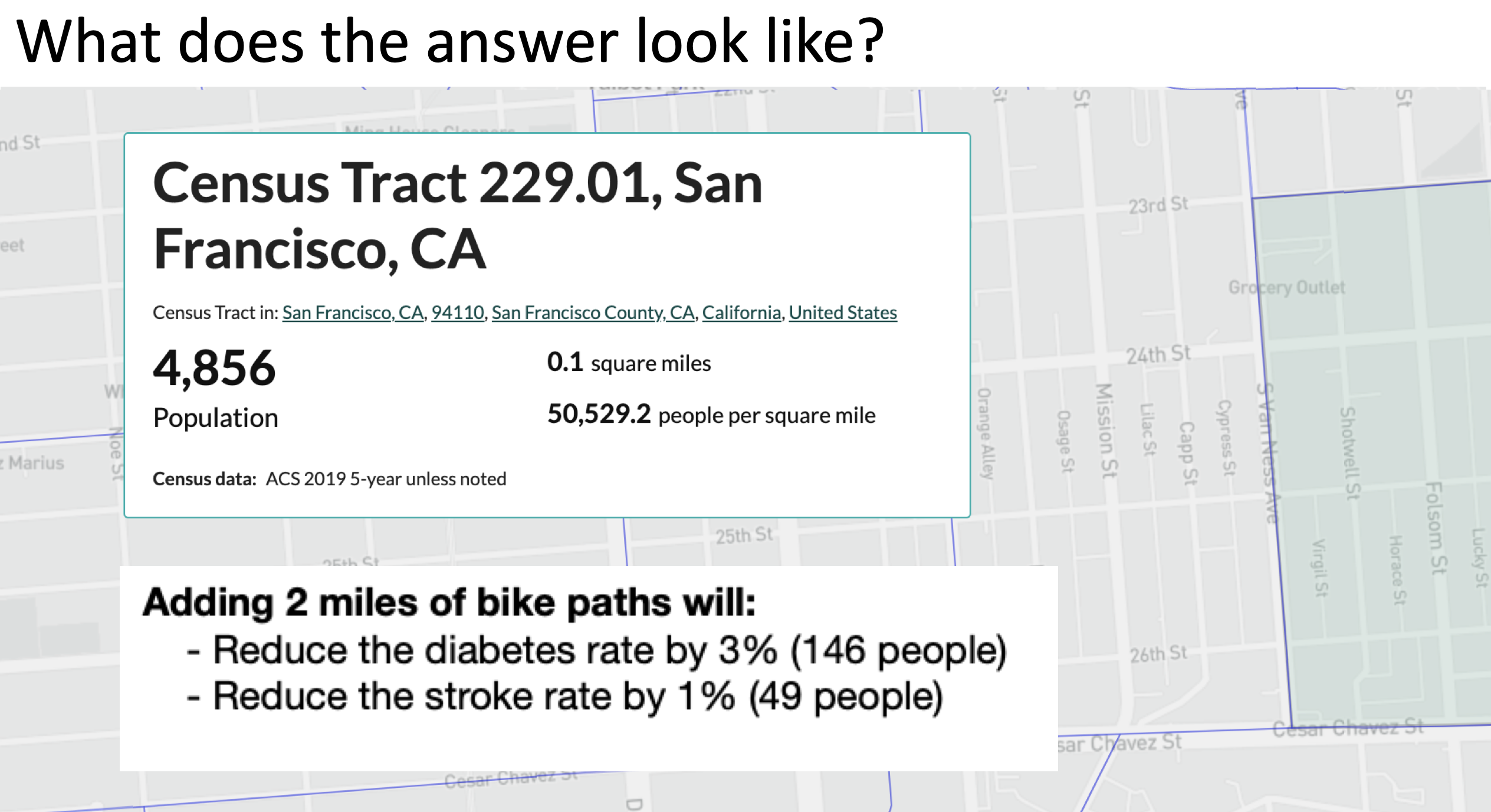

Supplement: Multimedia Appendix 9 [file publichealth_v8i8e37379_app9.zip › appendix_9/webapp/www/objective.png]
