# Supplementary material for: Estimating the Health Effects of Adding Bicycle and Pedestrian Paths at the Census Tract Level: Multiple Model Comparison
Source: JMIR Public Health Surveill. 2022 Aug 24;8(8):e37379. doi: 10.2196/37379 (PMC9453587; doi:10.2196/37379)
Supplement: Multimedia Appendix 8 [file publichealth_v8i8e37379_app8.zip › appendix_8/README.rtf]

This directory contains the recommended portfolio of BPPs that should be added to Norfolk using our described approach. It also contains a times series showing how those BPPs will manifest their improvements in health outcomes over a five year period.
